# Spatially non-overlapping Ca^2+^ signals drive distinct forms of neurotransmission

**DOI:** 10.1016/j.celrep.2023.113201

**Published:** 2023-09-30

**Authors:** Camille S. Wang, Lisa M. Monteggia, Ege T. Kavalali

**Affiliations:** 1 Vanderbilt Brain Institute, Vanderbilt University, Nashville, TN 3729-7933, USA; 2 Department of Pharmacology, Vanderbilt University, Nashville, TN 37240-7933, USA; 3 Lead contact

## Abstract

Calcium (Ca^2+^) signaling is tightly regulated within a presynaptic bouton. Here, we visualize Ca^2+^ signals within hippocampal presynaptic boutons using GCaMP8s tagged to synaptobrevin, a synaptic vesicle protein. We identify evoked presynaptic Ca^2+^ transients (ePreCTs) that derive from synchronized voltage-gated Ca^2+^ channel openings, spontaneous presynaptic Ca^2+^ transients (sPreCTs) that originate from ryanodine sensitive Ca^2+^ stores, and a baseline Ca^2+^ signal that arises from stochastic voltage-gated Ca^2+^ channel openings. We find that baseline Ca^2+^, but not sPreCTs, contributes to spontaneous glutamate release. We employ photobleaching as a use-dependent tool to probe nano-organization of Ca^2+^ signals and observe that all three occur in non-overlapping domains within the synapse at near-resting conditions. However, increased depolarization induces intermixing of these Ca^2+^ domains via both local and non-local synaptic vesicle turnover. Our findings reveal nanosegregation of Ca^2+^ signals within a presynaptic terminal that derive from multiple sources and in turn drive specific modes of neurotransmission.

## In brief

Wang et al. use GCaMP8s-Syb2 to visualize three distinct Ca^2+^ signals within individual presynaptic boutons, and these are derived from unique Ca^2+^ sources. Using an optical approach employing photobleaching as a use-dependent tool, they find these Ca^2+^ signals occur in spatially distinct domains and, furthermore, drive specific modes of neurotransmission.

## Graphical abstract

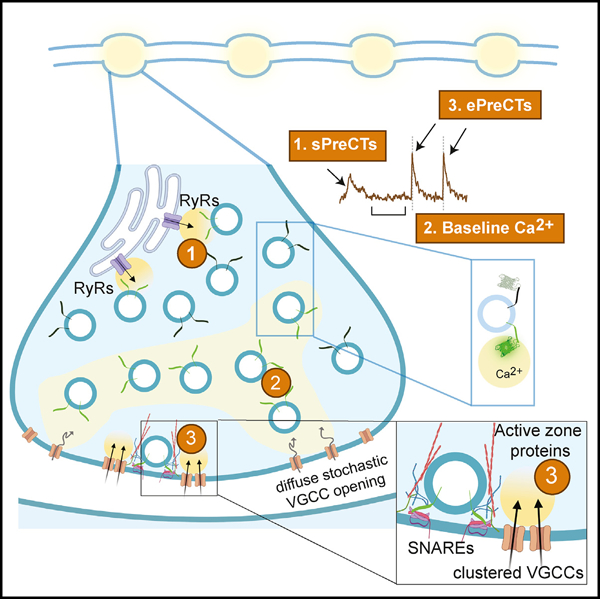

## INTRODUCTION

Ca^2+^ ions are a critical component of cellular physiology and play a key role in neurotransmission by triggering evoked neurotransmitter release and modulating spontaneous neurotransmission.^1,2^ Within a single synapse, Ca^2+^ signaling is very localized and tightly regulated. For instance, Ca^2+^ influx from voltage-gated Ca^2+^ channels (VGCCs) is limited to a “nanodomain” via buffering and efflux mechanisms.^3,4^ This rigorous control not only supports the regulation of intricate neuronal processes but also allows sub-synaptic specificity in signaling pathways. Within the presynaptic terminals alone, there are numerous sources of Ca^2+^ that regulate signaling pathways in addition to VGCCs, such as endoplasmic reticulum Ca^2+^ stores mediated by inositol triphosphate (IP3) and ryanodine receptors, mitochondrial Ca^2+^, and Ca^2+^ leak across the plasma membrane.^5–7^

Despite the essential role of Ca^2+^ in neurotransmission, the spatial organization of multiple Ca^2+^ sources within individual synapses is not fully understood. For instance, studies have found that distinct vesicle pools are affected by different Ca^2+^ sources to modulate synaptic transmission,^8,9^ while others have suggested that spatial location of vesicle pools does not affect their functional properties.^10^ Thus, the relationship between presynaptic Ca^2+^ sources and neurotransmission, as well as the spatial regulation of presynaptic Ca^2+^, remains to be elucidated.

Here, we investigated the spatial organization of distinct presynaptic Ca^2+^ sources using the Ca^2+^ sensing probe GCaMP8s tagged to synaptobrevin-2, a presynaptic synaptic vesicle protein (also called VAMP2), and we assessed their relationship to action potential evoked and spontaneous forms of neurotransmission. We identified three distinctly measurable Ca^2+^ signals—evoked presynaptic Ca^2+^ transients (ePreCTs), spontaneous presynaptic Ca^2+^ transients (sPreCTs), and baseline Ca^2+^ levels. Our examination showed that while, as expected, ePreCTs were due to synchronized VGCC openings, sPreCTs were derived from internal stores. Baseline Ca^2+^ levels were also partly driven by VGCCs, albeit in the form of putative asynchronous single-channel openings. We observed that spontaneous Ca^2+^ transients do not significantly contribute to spontaneous excitatory neurotransmission but rather that changes in baseline Ca^2+^ drive a significant fraction of this mode of release. Using photobleaching as a use-dependent blocker of GCaMP8s-Syb2 (synapsin promoter to drive expression of a GCaMP8s tagged to synaptobrevin-2) fluorescence,^11^ we showed that ePreCTs, sPreCTs, and baseline Ca^2+^ levels are detected by sensors on largely non-overlapping vesicle pools. We also found that while Ca^2+^ sources are spatially distinct at near-resting conditions, the Ca^2+^ sensor containing vesicle pools can intermix with increased activity.

Overall, we demonstrate that photobleaching of GCaMP8s-Syb2 can uncover spatially non-overlapping Ca^2+^ domains deriving from different sources within the presynaptic terminal and that these Ca^2+^ sources are specifically linked to distinct forms of neurotransmission.

## RESULTS

### Spontaneous and ePreCTs demonstrate distinct kinetics

**Figure 1.**
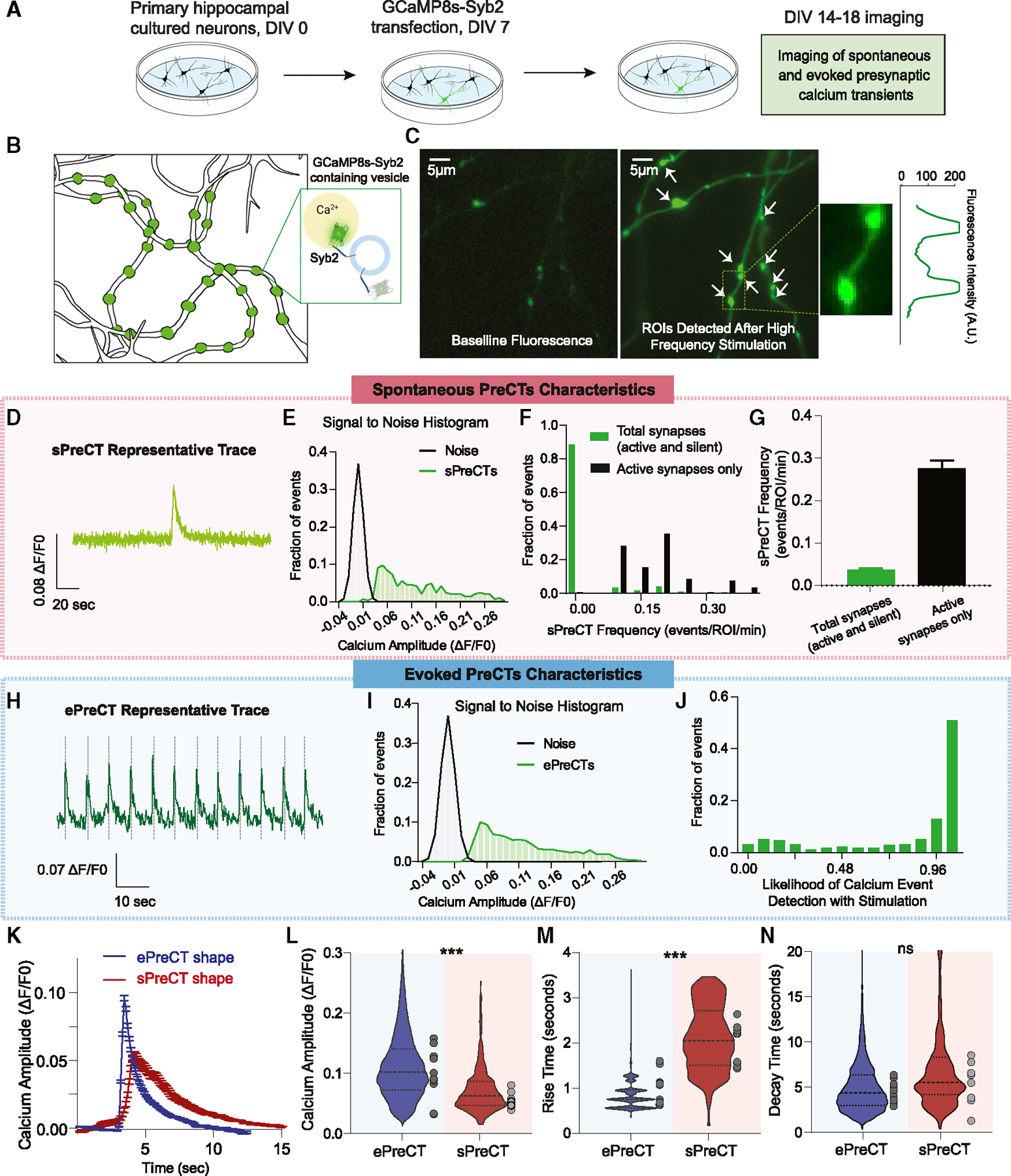
Kinetics of detected Ca^2+^ transients (A) Experimental design for calcium phosphate transfection of GCaMP8s-Syb2 in cultured hippocampal neurons. (B) Cartoon representation of GCaMP8s-Syb2 on presynaptic vesicles. (C) Representative image of neuronal synapses before and after high-frequency stimulation. (D) Representative spontaneous presynaptic Ca^2+^ transient (sPreCT) trace. (E) Histogram of detected spontaneous event amplitudes compared to the noise of the trace, from individual synapses (ROIs). (F) Histogram of sPreCT frequencies of all synapses (green) and active synapses only (black). n = 1,744 synapses. (G) Bar graph comparison of the sPreCT frequencies between all synapses versus active synapses only. n = 1,744 synapses. (H) Representative evoked presynaptic Ca^2+^ transient (ePreCT) trace. (I) Histogram of detected evoked event amplitudes compared to the noise of the trace, from individual synapses (ROIs). (J) Histogram distribution of the likelihood of detecting an ePreCT with each stimulation. n = 1,115 synapses. Note that the majority of synapses (~70%) respond to every stimulation with high fidelity (≥90%). (K) Averaged detected sPreCT and ePreCT events. (L–N) Comparison of the kinetics of ePreCT (N = 12 coverslips) versus sPreCT (N = 10 coverslips) in terms of Ca^2+^ amplitudes (L), rise time (M), and decay time (N). Welch’s t test. Graphs are mean ± SEM. Significance reported as ***p < 0.001. NS, non-significance.

To selectively detect Ca^2+^ signals within presynaptic terminals, we used the GCaMP8s-Syb2 construct. Primary hippocampal neurons were sparsely transfected with GCaMP8s-Syb2 and imaged at days *in vitro* (DIV) 15–18 (Figures 1A and 1B). Similar to earlier studies from our group,^11,12^ we delivered high-frequency stimulation or 90 mM K+ at the end of each experiment to identify active synapses. We then drew regions of interest (ROIs) over local maxima (Figure 1C) and measured their fluorescence activity over time from single synapses. These settings allow for visualization of fluorescent signals originating from individual boutons.^11^ Experiments were performed in the presence of 6-cyano-7-nitroquinoxaline-2,3-dione (CNQX) and (2R)-amino-5-phosphonovaleric acid (APV), which block -amino-3-hydroxy-5-methyl-4-isoxazolepropionic acid receptors (AMPARs) and N-methyl-D-aspartate receptors (NMDARs), respectively, to prevent recurrent neuronal activity. Prior work has demonstrated that these inhibitors are sufficient to inhibit significant contribution of spontaneous action potentials.^11^ Under these conditions, we isolated fluorescence responses originating from individual synaptic boutons.^11,13–15^

In the presence of these inhibitors, we detected sPreCTs with a high signal-to-noise ratio (Figures 1D and 1E). sPreCTs occur at a frequency of ~0.01–0.05 events/ROI/minute with ~90% of detected synapses being silent (Figures 1F and 1G). We detected evoked Ca^2+^ presynaptic transients with applied electrical stimulation (ePreCTs) (Figure 1H). Similar to sPreCTs, evoked events have a high signal-to-noise ratio (Figure 1I). We anticipated that each stimulation would lead to a conserved level of presynaptic Ca^2+^ influx,^1^ and indeed, we detected ePreCTs with every stimulation in the majority of synapses with a minimal number of failures, thus leading to a likelihood of detection close to 1 (Figure 1J). Of note within individual synapses (as plotted along the x axis), we detected substantial variability in amplitudes of Ca^2+^ signals among trials (Figure S1A), and this variability is not due to stimulator artifact (Figure S1B).

Though Ca^2+^ sensors may distort the endogenous Ca^2+^ signal due to their inherent buffering properties and kinetics,^16^ measuring the timing and shape of detected Ca^2+^ transients still provides useful information. The intensity of their fluorescence can provide insight into relative concentrations of presynaptic Ca^2+^. When we compared the shape of presynaptic Ca^2+^signals (Figure 1K), we found that sPreCTs have smaller amplitudes (Figure 1L), slower rise times (Figure 1M), and similar decay times (Figure 1N) compared with ePreCTs. When external Ca^2+^ concentrations were increased, ePreCT amplitudes increased (Figures S2A–S2C). sPreCT frequencies and amplitudes are comparable in 2 versus 8 mM Ca^2+^, suggesting that sPreCTs are not directly affected by external Ca^2+^ concentrations (Figures S2D–S2F).

### Evoked Ca^2+^ transients derive from VGCCs

**Figure 2.**
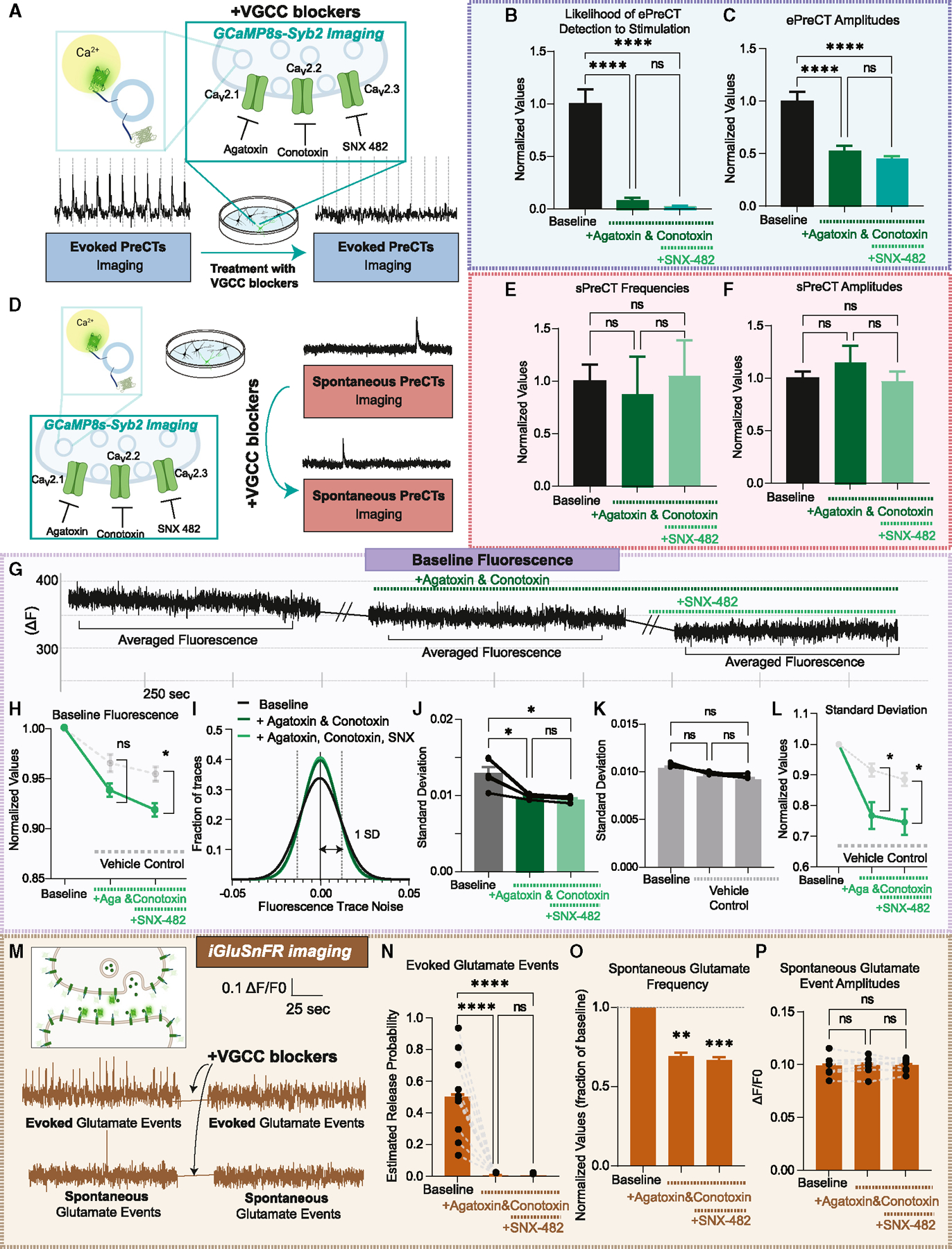
Effects of blocking VGCCs on presynaptic Ca^2+^ and excitatory neurotransmission (A) Experimental design and representative traces of VGCC blockade on ePreCTs. (B and C) Effects of VGCC blockade on ePreCT likelihood to stimulation (B) and amplitudes (C). N = 9 coverslips. One-way ANOVA. (D) Experimental design and representative traces of VGCC blockade on sPreCTs. (E and F) Effects of VGCC blockade on sPreCT frequencies (E) and amplitudes (F). Normalized values for GCaMP8s-Syb2 imaging were calculated by dividing each synaptic value after treatment by the average of the coverslip during before conditions. sPreCT frequencies were so infrequent that we could not normalize synapse by synapse without losing information; thus, we had to normalize this way, and we kept it consistent across other measurements in this experiment. N = 9 coverslips. One-way ANOVA was performed for these experiments. (G) Representative traces of baseline Ca^2+^ before and after VGCC blockade. (H) Changes in baseline fluorescence after VGCC blockade compared with controls. N = 5 coverslips for treatment groups; N = 3 coverslips for control groups. Two-way ANOVA. (I) Frequency distribution of the noise of the trace before and after treatment, with the standard deviation demarcated by dotted lines. (J) Comparison of the standard deviation of the trace after VGCC blockade. One-way ANOVA. (K) Comparison of the standard deviation of the trace after vehicle control addition. One-way ANOVA. (L) Comparison of standard deviation after vehicle control compared with after VGCC blockade. Two-way ANOVA. (M) Representative iGluSnFR traces before and after VGCC blockade. (N–P) Effect of VGCC blockade on glutamate evoked release probability (N), spontaneous glutamate frequency (O), and amplitudes (P). Normalized values for iGluSnFR were made by normalizing synapse by synapse such that all normalized “before” values are 1. N = 10 coverslips for evoked glutamate events; N =7 coverslips for spontaneous glutamate events. One-way ANOVA was performed on these experiments. Graphs are mean ± SEM. Significance reported as *p < 0.05, **p < 0.01, ***p < 0.001, and ****p < 0.0001. NS, non-significance.

We next sought to identify the source from which ePreCTs arise. As ePreCTs occur in response to stimulation, these Ca^2+^ influxes are likely mediated by VGCCs. VGCCs are positioned within nanometers of a docked and primed vesicle such that Ca^2+^ entering through these ion channels binds to synaptotagmin-1 and induces rapid vesicle fusion.^1,17^ There are several VGCCs sub-types at presynaptic neuronal terminals,^18,19^ and each channel type can be blocked with specific toxins. To measure VGCC contributions toward ePreCTs, we used Ω-Agatoxin IVA to block Ca_v_2.1 Ca^2+^ channels (P/Q type),^20,21^ ω-Conotoxin GVIA to block Ca_v_2.2 Ca^2+^ channels (N-type),^22–24^ and SNX 482 to block Ca_v_2.3 Ca^2+^ channels (R-type) (Figure 2A).^25^

The block of Ca_v_2.1 and Ca_v_2.2 Ca^2+^ channels causes a 95% decrease in ePreCTs and an up to ~98% decrease with the additional blockage of Ca_v_2.3 Ca^2+^ channels (Figure 2B). Furthermore, the amplitudes of any residually detected ePreCTs are significantly decreased (Figure 2C). These results suggest that VGCCs are the primary source of ePreCTs. Surprisingly, VGCC blockers did not affect sPreCTs (Figures 2D–2F), suggesting that sPreCTs are likely derived from a different source.

We next measured the baseline Ca^2+^ signal by averaging the fluorescence of a single synapse during a time of minimal to no Ca^2+^ transients, which reflect a more global Ca^2+^ level in the synapse (Figure 2G). We find that VGCC blockers significantly decrease baseline Ca^2+^ compared with control groups (Figure 2H). We also recorded baseline Ca^2+^ signals with similar imaging times without any toxins to account for any unintended photobleaching or decay of the baseline signal. We detected a decrease in the standard deviation of the baseline Ca^2+^ noise within the traces before and after addition of toxins, consistent with inhibition of baseline signal rather than passive decay (Figure 2I). A genuine reduction in the baseline Ca^2+^ signal is reflected as a decrease in fluorescent trace’s absolute value and the standard deviation of its noise. We find that VGCC blockers significantly decrease the noise of the trace compared with the control conditions (Figures 2J–2L). These results support that VGCC inhibition leads to a decrease in baseline Ca^2+^.

We investigated whether this baseline Ca^2+^ change has any functional effects on synaptic transmission using a glutamate sensing probe, iGluSnFR (Figure 2M).^11,26,27^ As we previously validated in a recent study, to monitor glutamatergic synapses, we drew ROIs around puncta that responded to high-frequency stimulation or elevated K^+^.^11^ As expected, evoked glutamate release events are completely abolished with the addition of VGCC blockers (Figure 2N). Furthermore, the initial estimated release probability values are consistent with the premise that we are measuring signals originating from single synapses.^11,28,29^ As for spontaneous glutamate events, we find that the addition of Ω-Agatoxin IVA and ω-Conotoxin GVIA significantly decreases spontaneous glutamate event frequency to 69% of its baseline values. The addition of SNX 482 causes a further decrease to 67% of baseline values (Figure 2O), demonstrating that presynaptic VGCCs contribute to over 30% of spontaneous glutamate release. The amplitudes of spontaneous glutamate events are similar between initial and treated conditions, consistent with their quantal nature (Figure 2P). Taken together, these results suggest that VGCCs not only drive evoked vesicle fusion but also contribute to spontaneous fusion by affecting resting baseline Ca^2+^ signaling.

### Ryanodine receptors on internal Ca^2+^ stores gate sPreCT occurrence

**Figure 3.**
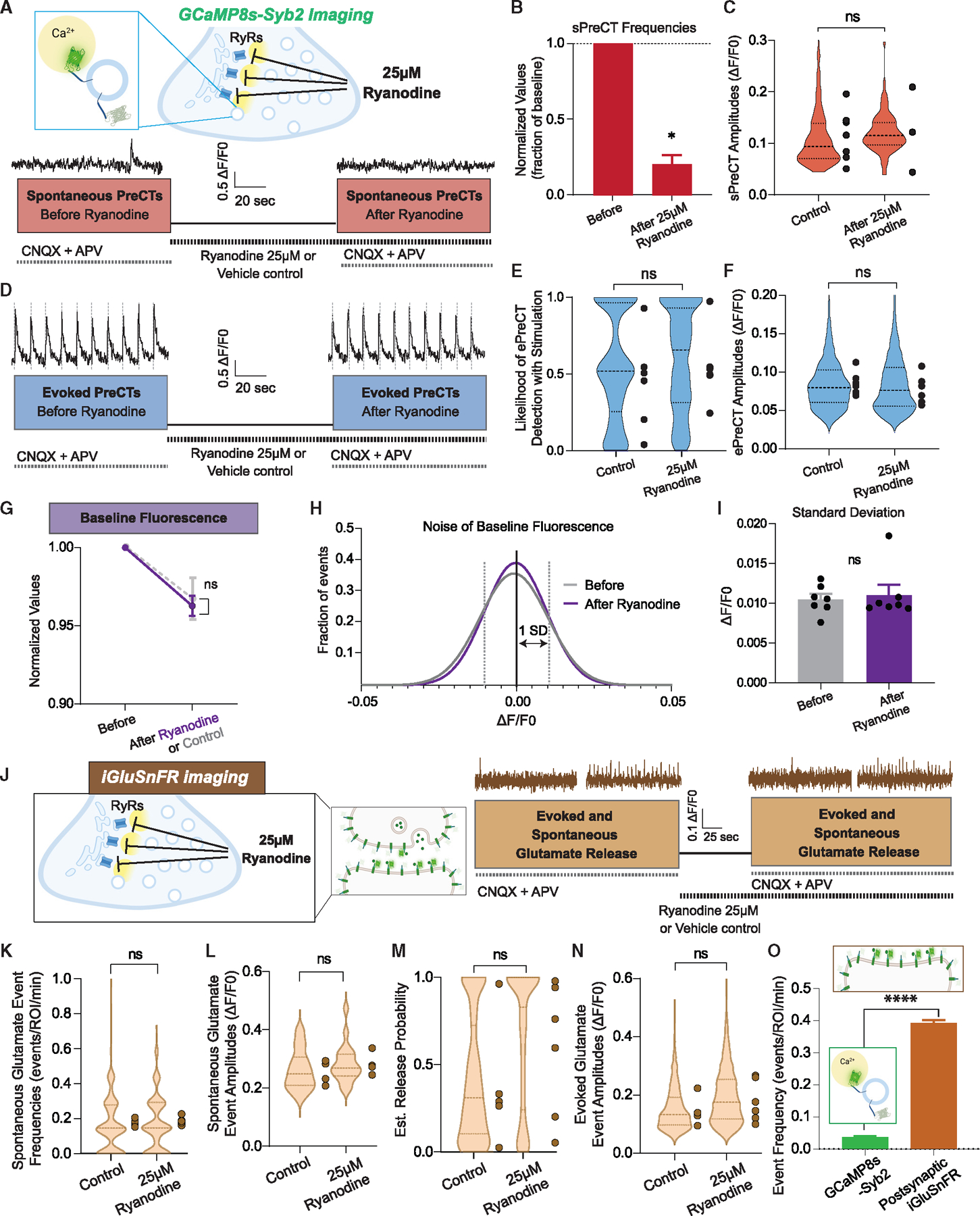
Ryanodine inhibition on presynaptic Ca^2+^ and glutamate release (A) Experimental design of ryanodine on spontaneous PreCTs with representative traces. (B and C) Effect of ryanodine inhibition on sPreCT frequencies (B) and amplitudes (C). N = 7 coverslips. Welch’s t test. (D) Experimental design of ryanodine on evoked PreCTs with representative traces. (E and F) Effect of ryanodine receptor inhibition on ePreCT likelihood to stimulation (E) and ePreCT amplitudes (F). N = 7 coverslips. Welch’s t test. Note that DMSO treatment by itself reduces the likelihood of Ca^2+^responses to stimulation. (G) Effect of ryanodine inhibition on baseline Ca^2+^ fluorescence. N = 7 coverslips for treatment group; N = 6 coverslips for control group. Two-way ANOVA. (H) Frequency distribution of the noise of the trace before and after ryanodine inhibition, with the standard deviation demarcated by dotted lines. (I) Comparison of the standard deviation of the fluorescent trace before and after ryanodine inhibition. N = 7 coverslips. Welch’s t test. (J) Experimental paradigm of ryanodine on glutamate release via iGluSnFR recordings, with representative traces. (K–N) Effect of ryanodine inhibition on spontaneous glutamate event frequencies (K), spontaneous glutamate event amplitudes (L), evoked release probability (M), and evoked glutamate event amplitudes (N). N = 7 coverslips for treatment group; N = 6 coverslips for control group. Welch’s t test. (O) Comparison of spontaneous presynaptic Ca^2+^ event frequencies with spontaneous glutamate event frequencies. N = 1,844 synapses for sPreCTs; n = 2,896 synapses for spontaneous glutamate events. Welch’s t test. Graphs are mean ± SEM. Significance reported as *p < 0.05. ****p < 0.0001. NS, non-significance.

A likely candidate source for sPreCTs is internal Ca^2+^ stores, which are mediated by IP3 and ryanodine receptors on the endoplasmic reticulum.^5,30,31^ At high concentrations, ryanodine blocks ryanodine receptors in a use-dependent manner.^32,33^ To test whether ryanodine-gated Ca^2+^ stores contribute to sPreCTs, we measured initial Ca^2+^ signals in the presence of activity-blocking drugs, followed by a perfusion of 25 μm ryanodine without any other drugs to facilitate the blockade of all ryanodine receptors. We then measured Ca^2+^ signals in ryanodine with activity-blocking drugs re-added (Figure 3A).

We find that ryanodine application significantly decreases sPreCT frequencies to 21% of initial values, though sPreCT amplitudes remain unaffected (Figures 3B and 3C). Ryanodine does not affect ePreCTs compared with a DMSO control (Figures 3D–3F), suggesting that internal Ca^2+^ transients driven by ryanodine receptors do not significantly contribute to ePreCTs. When examining baseline Ca^2+^, ryanodine application does not significantly lower baseline fluorescence compared with the control groups (Figure 3G), and the standard deviation of the fluorescent trace is unchanged as well (Figures 3H and 3I). Furthermore, ryanodine does not affect evoked nor spontaneous glutamate events as measured by iGluSnFR (Figure 3J–3N). Other studies using electrophysiology have also found that ryanodine does not block mEPSCs.^29,34^ These results suggest that sPreCTs derive largely from internal stores gated by ryanodine receptors and that ryanodine receptor opening does not significantly contribute to spontaneous glutamate events under resting conditions. In agreement with this premise, we find that caffeine, which is known to increase miniature excitatory postsynaptic current (mEPSC) frequency,^35^ increases the baseline Ca^2+^ signal (Figure S3A–S3C). Caffeine application also occludes sPreCT frequency, presumably via depleting ryanodine-sensitive internal Ca^2+^ stores (Figure S3D and S3E), indicating that sources of sPreCTs and caffeine-induced Ca^2+^ release overlap, although individual sPreCTs do not significantly contribute to spontaneous glutamate events. Finally, we observed that sPreCT frequencies are an order of magnitude lower than spontaneous glutamate event frequencies measured with both iGluSnFR (Figure 3O) and electrophysiology.^36,37^ As a large majority of spontaneous glutamate events do not correspond with the occurrence of spontaneous Ca^2+^ transients, this result further supports the notion that sPreCTs are not a major contributor to spontaneous glutamate release.

### Evoked and spontaneous Ca^2+^ transients are photobleached independently

**Figure 4.**
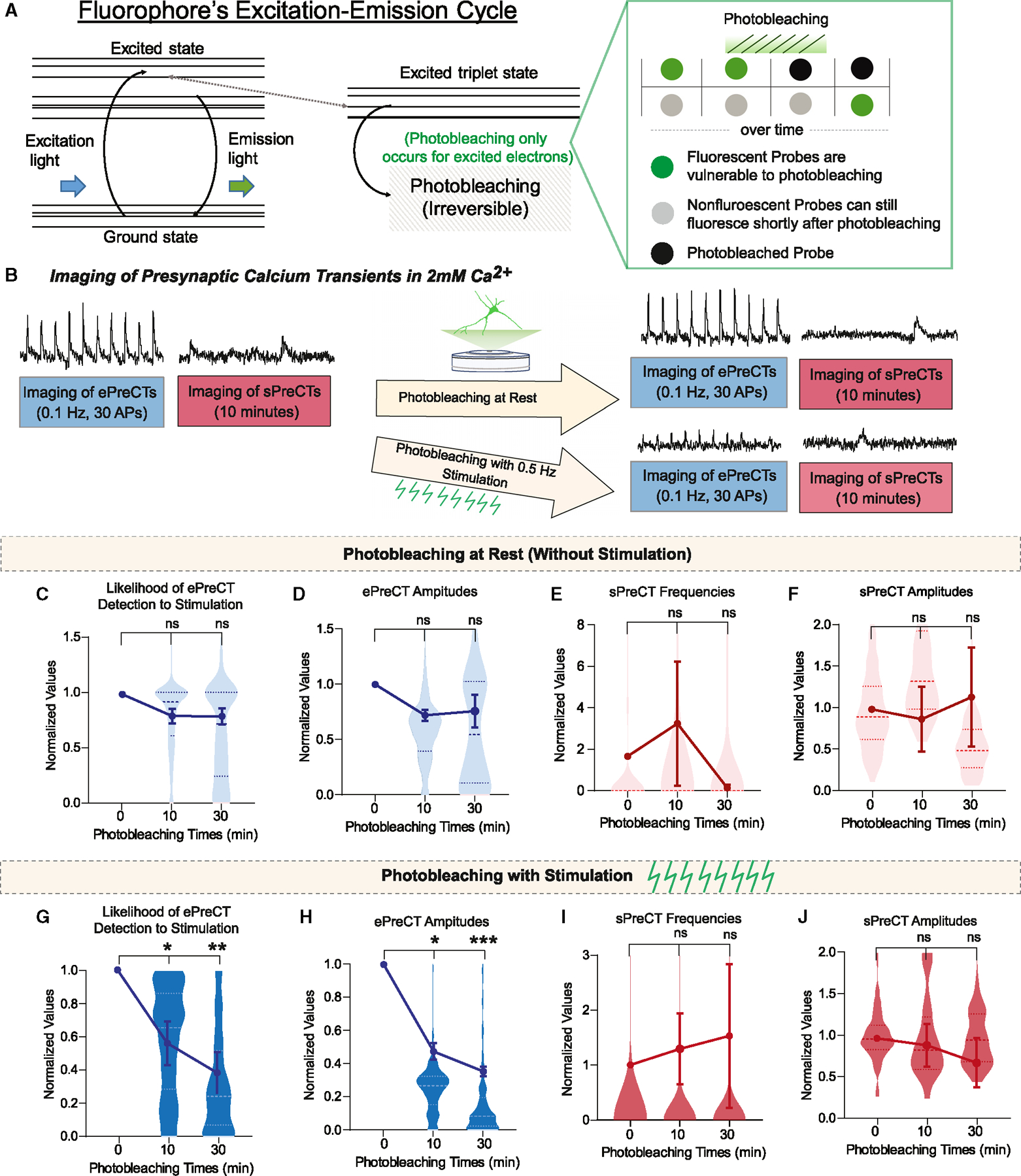
Effect of photobleaching on ePreCT and sPreCTs (A) Photobleaching schematic on its use-dependent property. (B) Experimental paradigm of photobleaching presynaptic Ca^2+^ transients at rest or with stimulation. (C and D) Effect of photobleaching at rest on ePreCT event likelihood to stimulation (C) and ePreCT amplitudes (D). N = 4 coverslips per group. One-way ANOVA. (E and F) Effect of photobleaching at rest on sPreCT frequencies (E) or sPreCT amplitudes (F). N = 6 coverslips for 10 min of photobleaching; N = 7 coverslips at 30 min of photobleaching. One-way ANOVA. (G and H) Effect of photobleaching with stimulation on ePreCT event likelihood to stimulation (G) and ePreCT amplitudes (H). N = 5 coverslips for all groups. One-way ANOVA. (I and J) Effect of photobleaching with stimulation on sPreCT frequencies (I) or sPreCT amplitudes (J). N = 4 coverslips for all groups. One-way ANOVA. Graphs are mean ± SEM. Significance reported as *p < 0.05, **p < 0.01, and ***p < 0.001. NS, non-significance.

In a previous study, we demonstrated the application of photobleaching as a use-dependent inhibitor of fluorescence to probe sub-synaptic spatial organization of spontaneous and evoked release.^11^ Photobleaching is use dependent, as only probes that are fluorescent (in the excited state) can be photobleached, and non-fluorescent probes in the ground state are unaffected by photobleaching (Figure 4A).^38^ We used this use-dependent property of photobleaching to examine the spatial organization of Ca^2+^ signals within the presynaptic terminal.

After obtaining initial measurements of presynaptic Ca^2+^ transients, we photobleached the entire field of view with maximal-intensity illumination and then resumed normal imaging of Ca^2+^ events from the photobleached synapses (Figure 4B). First, we photobleached at rest, in which photobleaching occurred without any applied stimulation. Under these conditions, neither ePreCTs nor sPreCTs are affected even up to 30 min of photobleaching at rest (Figures 4C–4F). However, when photobleaching was applied with stimulation (Figure 4B), the ePreCT fluorescence is significantly photobleached, while sPreCTs remain unaffected (Figures 4G–4J). These results demonstrate that photobleaching at rest versus with stimulation has a differential effect on ePreCTs, consistent with the premise that Ca^2+^ probes that sense ePreCTs are selectively activated by stimulation, thus rendering them susceptible to photobleaching. Moreover, the resistance of sPreCTs to photobleaching may be related to their low frequency. Overall, the differential effects of photobleaching suggest that ePreCTs and sPrCTs occur in spatially distinct, non-overlapping domains within the presynaptic terminal.

### Ca^2+^ domains intermix with increased activity

**Figure 5.**
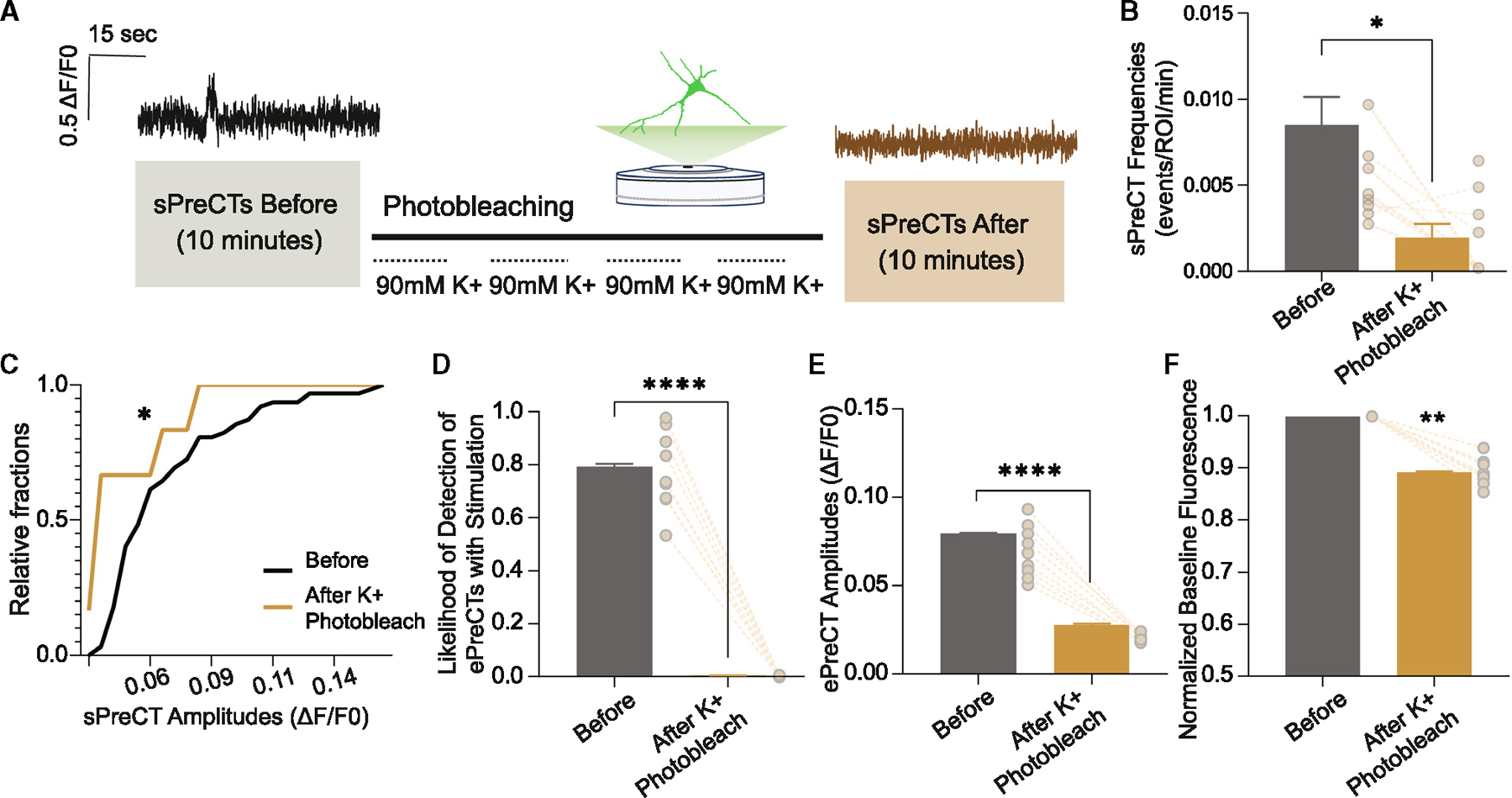
Effect of photobleaching with high potassium perfusion (A) Experimental paradigm. (B and C) Effect of perfusing high K^+^ while photobleaching on sPreCT frequencies (B) and sPreCT amplitudes (C). N = 8 coverslips. Welch’s t test for (B) and Kolmogorov-Smirnov test for (C). (D–F) Effect of perfusing high K^+^ while photobleaching on ePreCT likelihood to stimulation (D), ePreCT amplitudes (E), and baseline Ca^2+^ signal (F). N = 9 coverslips. Welch’s t test. Graphs are mean ± SEM. Significance reported as *p < 0.05, **p < 0.01, and ****p < 0.0001. NS, non-significance.

We examined whether saturating presynaptic milieu with Ca^2+^ would make sensors detecting sPreCTs sensitive to photobleaching. For this purpose, we applied 90 mM K^+^ at 2 min intervals over the course of 30 min (Figure 4A), which is expected to photobleach all available Ca^2+^ signals including the difficult-to-photobleach sPreCTs. With 90 mM K^+^ perfusion over the course of photobleaching, we were indeed able to significantly decrease the detection of sPreCTs in terms of their frequencies, as well as their amplitudes (Figures 4B and 4C). We find that ePreCTs are significantly affected by photobleaching with elevated K^+^ (Figures 5D and 5E), and baseline Ca^2+^ was also significantly affected (Figure 5F). These results suggest that elevated K^+^ stimulation elicits a large and generalized activity that recruits otherwise infrequently activated Ca^2+^ sensors, rendering them sensitive to photobleaching. Elevated K^+^ conditions could also broaden Ca^2+^ domains from discrete to overlapping regions, which would further enhance photobleaching effects on different Ca^2+^ signals.

### Baseline Ca^2+^ levels are sensitive to photobleaching

**Figure 6.**
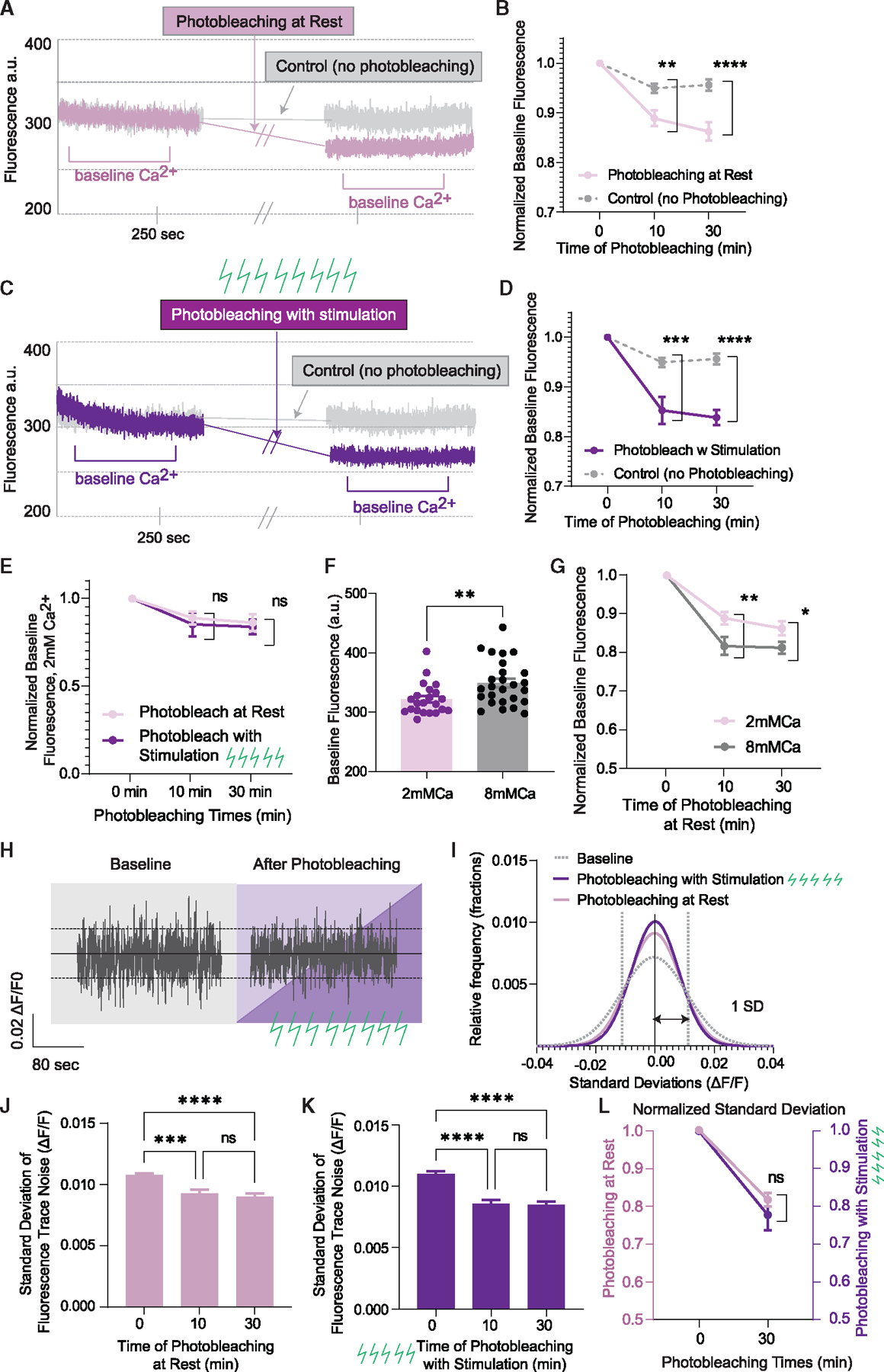
Effect of photobleaching on baseline Ca^2+^ (A) Experimental paradigm of photobleaching at rest on baseline Ca^2+^ signal. (B) Photobleaching at rest on baseline Ca^2+^. N = 6 coverslips for 10 min; N = 7 for 30 min of photobleaching. Two-way ANOVA. (C) Experimental paradigm of photobleaching with stimulation on baseline Ca^2+^. (D) Photobleaching with stimulation on baseline Ca^2+^. N = 6 coverslips for 10 min; N = 7 for 30 min of photobleaching. The same control baseline group was used as for (B). Two-way ANOVA. (E) Comparison of the effect of photobleaching at rest versus with stimulation on baseline Ca^2+^ signal. N = 6 coverslips for 10 min. N = 7 for 30 min of photobleaching per group. Two-way ANOVA. (F) Comparison of baseline Ca^2+^ signal in 2 versus 8 mM Ca^2+^. N = 23 coverslips for 2 mM and N = 27 coverslips for 8 mM Ca^2+^. Welch’s t test. (G) Comparison of photobleaching on baseline Ca^2+^ signal in 2mM Ca^2+^ (N = 6 coverslips for 10 min, N = 7 for 30 min of photobleaching) versus 8 mM Ca^2+^ (N = 7 coverslips for 10 min, N = 6 for 30 min of photobleaching). Two-way ANOVA. (H) Representative baseline Ca^2+^ signals before and after photobleaching. (I) Frequency distribution of the noise of the trace before and after photobleaching. (J and K) Standard deviation of the fluorescent trace before and after photobleaching at rest (N = 5 coverslips for 10 min, N = 6 for 30 min of photobleaching) (J) and with stimulation (N = 6 coverslips for 10 min, N = 7 for 30 min of photobleaching) (K). One-way ANOVA. (L) Comparison of the standard deviations between photobleaching for 30 min at rest (N = 6) and with stimulation (N = 7). Two-way ANOVA. Graphs are mean ± SEM. Significance reported as *p < 0.05, **p < 0.01, ***p < 0.001, and ****p < 0.0001. NS, non-significance.

We next asked if sPreCTs and baseline Ca^2+^ signals derive from different Ca^2+^ sources, then do they also occur in spatially non-overlapping domains? To investigate this, we measured baseline Ca^2+^ before and after photobleaching at rest (Figure 6A). We find that baseline Ca^2+^ is significantly affected by photobleaching at rest within 10 min and even more so after 30 min (Figure 6B). When photobleached with stimulation, baseline Ca^2+^ was also decreased (Figures 6C and 6D). Comparing the rate of photobleaching at rest versus with stimulation on baseline Ca^2+^, we found that there is no significant difference between these two conditions (Figure 6E). This result suggests that the photobleaching effect on baseline Ca^2+^ signal is saturated by photobleaching under resting conditions such that the application of stimulation does not induce further photobleaching. Furthermore, because stimulating while photobleaching does not further decrease the baseline Ca^2+^ signal as it does to ePreCTs, these results suggest that Ca^2+^ sensors that detect baseline Ca^2+^ are distinct from Ca^2+^ sensors that detect ePreCTs.

We observed that baseline Ca^2+^ signals were increased in the presence of 8 mM Ca^2+^ (Figure 6F). Specifically, there was increased sensitivity to photobleaching in higher extracellular Ca^2+^, as more probes detecting baseline Ca^2+^ signals are fluorescent and thus available to be photobleached (Figure 6G). Though baseline Ca^2+^ signals are more vulnerable to photobleaching in higher external Ca^2+^ concentrations, stimulation during photobleaching does not further affect the photobleaching rate, similar to more physiological concentrations of Ca^2+^ (Figure S4C). Furthermore, the standard deviation of the fluorescence trace was decreased by photobleaching at rest and with stimulation to the same degree (Figure 6H–6L). The decrease in standard deviation of the trace further supports that baseline Ca^2+^ is being photobleached and that the decrease in fluorescence is not due to passive decay.

### Recovery from photobleaching is facilitated by activity

**Figure 7.**
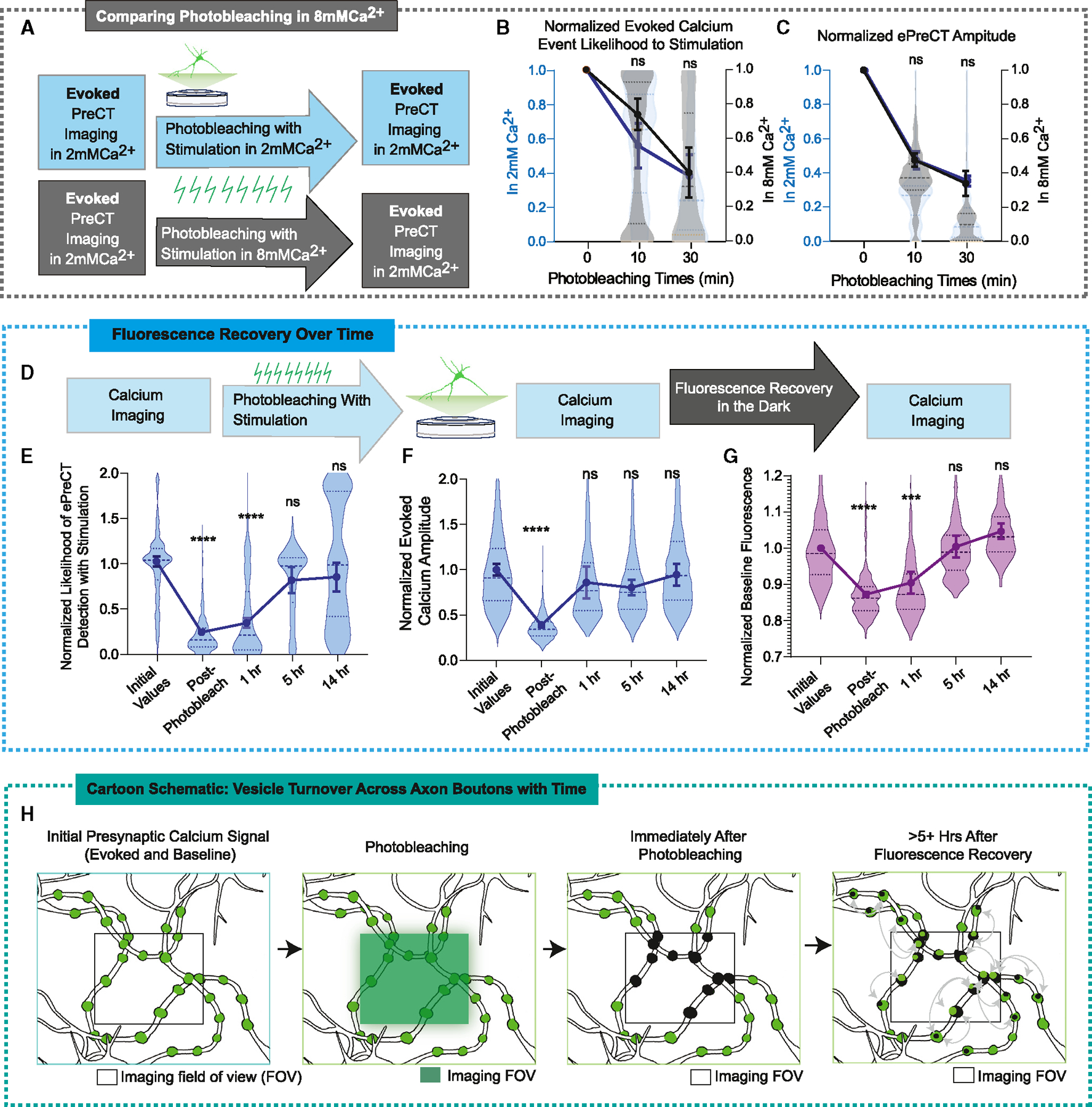
Intermixing of vesicle pools occurs with time and with activity (A) Experimental paradigm of photobleaching in 8 mM Ca^2+^. (B) Comparison of photobleaching with stimulation on ePreCT likelihood to stimulation in 2 (N = 4 coverslips for 10 min, N = 5 for 30 min of photobleaching) versus 8 mM Ca^2+^ (N = 7 coverslips for 10 min, N = 6 for 30 min of photobleaching). Two-way ANOVA. (C) Comparison of photobleaching with stimulation on ePreCT amplitudes in 2 (N = 4 coverslips for 10 min, N = 4 for 30 min of photobleaching) versus 8 mM Ca^2+^ (N = 7 coverslips for 10 min, N = 6 for 30 min of photobleaching). Two-way ANOVA. (D) Experimental paradigm of fluorescence recovery over time. (E–G) Effect of fluorescence recovery over time on ePreCT likelihood to stimulation (E), ePreCT amplitudes (F), and baseline Ca^2+^ signal (G). N = 5 coverslips at 1 h of photobleaching; N = 6 coverslips for 5 h; N = 5 coverslips for 14 h. One-way ANOVA. (H) Cartoon schematic of local and non-local vesicle turnover and subsequent fluorescence recovery over time after photobleaching. Graphs are mean ± SEM. Significance reported as *p < 0.05, **p < 0.01, ***p < 0.001, and ****p < 0.0001. NS, non-significance.

We next sought to determine whether we could alter the effect of photobleaching on Ca^2+^ transients. Increasing the external concentration of Ca^2+^ increases its concentration gradient at the neuronal membrane such that with each VGCC opening, a greater amount of Ca^2+^ flows into the intracellular space (Figures S2A–S2C). We hypothesize that as more fluorescent probes are active with each stimulation, they will be more readily available to be photobleached and thus have an increased rate of photobleaching in 8 mM Ca^2+^. However, photobleaching at higher Ca^2+^ concentrations did not significantly alter the rate of ePreCT photobleaching (Figures 7A–7C and S5). These data are in contrast to the increased degree of photobleaching of baseline Ca^2+^ signals in elevated extracellular Ca^2+^. We surmised that these results may in part be due to increased mixing of unbleached fluorescent probes into the bleached region, thus augmenting the rate of fluorescence recovery during photobleaching.

To address the extent of diffusion between bleached and unbleached GCAMP8-Syb2 probes, we next measured the fluorescence rate of recovery after extensive photobleaching. In 2 mM Ca^2+^, we photobleached while stimulating for 30 min to ensure maximal photobleaching and then allowed fluorescence recovery for 30 min in the dark (Figure S4D). Under these conditions, we did not observe any significant recovery of ePreCTs (Figure S4E). We sought to examine whether we could increase the rate of fluorescence recovery by augmenting vesicle recycling via increased neuronal activity. The application of elevated K^+^ or stimulation during the fluorescence recovery phase did not lead to a substantial increase in the rate of fluorescence recovery (Figures S4K–S4M), indicating that activity is not critical in facilitating recovery of the GCAMP8-Syb2 pool.

As ePreCTs can be photobleached, we next queried whether they can also recover their fluorescence with time. Thus, we photobleached ePreCTs for 30 min with stimulation to maximize the photobleaching effect and then measured ePreCTs after multiple time points of recovery in the dark (Figure 7D). We found that ePreCTs recover over the course of hours, and by 5 h of recovery, ePreCT detection returns to original levels prior to photobleaching (Figures 7E and 7F). The fluorescence recovery of baseline Ca^2+^ also occurs after several hours of recovery in the dark (Figure 7G).

## DISCUSSION

Ca^2+^ ions play an essential role in synaptic physiology, and at presynaptic terminals, they impact multiple forms of neurotransmitter release. However, the nano-organization of distinct presynaptic Ca^2+^ sources and their specific effect on different modes of neurotransmission within individual boutons remain poorly understood. We examined this problem in hippocampal neurons using GCaMP8s-Syb2 to detect three sources of Ca^2+^: ePreCTs, sPreCTs, and baseline Ca^2+^. We observed that these three different Ca^2+^ signals derive from distinct sources and enact specific effects on neurotransmission (Table 1). Photobleaching experiments revealed these Ca^2+^ signals occur in spatially distinct and non-overlapping sub-synaptic domains. Increased neuronal activity, such as applied stimulation or elevated K^+^, showed the spatial and functional domains of these Ca^2+^ signals beginning to overlap. Taken together, these findings are consistent with the premise that Ca^2+^ signals are tightly coordinated in their spatial domains within a single synapse.

### The relationship between presynaptic Ca^2+^ and spontaneous glutamate fusion

Presynaptic Ca^2+^ levels are a critical modulator of spontaneous neurotransmitter release.^39–41^ Still, the exact nature of this relationship remains an unanswered question.^2^ Internal Ca^2+^ stores are a possible source linking Ca^2+^ to spontaneous fusion.^42–45^ We employed both GCaMP and iGluSnFR and observed under resting conditions that ryanodine-sensitive Ca^2+^ stores do not contribute to spontaneous glutamate release at near-resting conditions. However, we cannot exclude a small effect that may be lost within the inherent variability of spontaneous release measurements. We also find that baseline Ca^2+^ is not affected by blocking ryanodine receptors but is decreased by inhibition of VGCCs. The functional role of VGCCs on spontaneous glutamate release remains controversial, with differing results reported within similar systems.^46–51^ In our study, we find that the blockade of Ca_v_2.1–3 Ca^2+^ channels significantly inhibits spontaneous excitatory fusion. While other studies have used Cd^2+^ to examine VGCC contribution toward neurotransmission, we did not use Cd^2+^ because application in our hands causes non-specific fluorescence signals.^52,53^ Additionally, we cannot exclude the possibility that L-type calcium channels may also contribute to neurotransmitter release.^54^

These results demonstrate that baseline Ca^2+^, but not sPreCTs, provides the link between presynaptic Ca^2+^ and spontaneous glutamate release. Our measurement suggests that stochastic single-channel openings, rather than coordinated multichannel openings, of VGCCs are in part responsible for the baseline Ca^2+^ signals. Thus, rather than detecting Ca^2+^ “transients,” we see a baseline shift in fluorescence due to the uncoordinated manner of single-channel openings across the presynaptic terminal. As baseline Ca^2+^ and ePreCTs occur in spatially distinct regions, VGCCs that contribute to spontaneous glutamate release are likely distributed outside of nanodomain surrounding sites of evoked release. In contrast, under the same conditions, ryanodine-sensitive stores do not appear to significantly contribute to this signal but rather may contribute toward other cellular processes.^55^ Furthermore, a decrease in baseline Ca^2+^ correlated with a decrease in spontaneous glutamate release, suggesting that baseline Ca^2+^ is a key regulator of spontaneous glutamate release.

Under the same conditions, spontaneously occurring Ca^2+^ transients appear to originate from ryanodine-sensitive Ca^2+^ stores. While the accuracy of detecting ryanodine receptor activation may be limited due to indirect coupling between our Ca^2+^ sensors and the location of Ca^2+^ stores, our main conclusion is that ryanodine receptor block decreases sPreCTs but does not affect any other Ca^2+^ or glutamate parameter we measured.

### Detected Ca^2+^ sources are spatially distinct at rest

To understand the spatial organization of these different modes of Ca^2+^ signaling, we used photobleaching as a use dependent inhibitor of fluorescence. With photobleaching at rest, neither spontaneous nor evoked preCTs were affected, but baseline Ca^2+^ was significantly photobleached. After photobleaching with stimulation, we found that ePreCTs were susceptible, while sPreCTs were not. While sensors detecting ePreCTs only become available to be photobleached during stimulation, this did not affect the rate at which baseline Ca^2+^ is photobleached, suggesting that ePreCTs, sPreCTs, and baseline Ca^2+^ sensors occupy non-overlapping vesicle pools. Using photobleaching, we find that the three measurable Ca^2+^ sources occur in spatially distinct vesicular domains at the presynaptic terminal. These results are consistent with earlier studies suggesting that functionally distinct vesicle pools occur in spatially different regions within the presynaptic terminal.^8,9,56^ Furthermore, elevated K^+^ induced depolarization and Ca^2+^ influx enabled intermixing of otherwise non-overlapping spatial domains and led to photobleaching of all detectable Ca^2+^ signals.

### Detectable Ca^2+^ sources intermix with time and increased activity

Recent studies have demonstrated the presence of a vesicle “superpool” that spans multiple terminals where vesicles are highly mobile and rapidly exchanged not only within a synapse but across multiple presynaptic terminals. Using a photoswitchable fluorochrome, Staras et al. observed that vesicles turn over at ~4% of the total pool per minute, and within an hour, there is clear fluorophore exchange across distant and neighboring synapses.^57^ In our study, we tested fluorescence recovery after photobleaching, which allows us to quantify the rate of vesicle movement from unbleached regions into the photobleached ones. Within a few hours, there was a complete recovery of ePreCT similar to the time course of superpool movement reported by Staras and colleagues (Figure 7K). This suggests that the recovery of probe fluorescence over time may be due to local and non-local vesicle turnover. In our study, we found that the effect of activity on fluorescence recovery (as a proxy for vesicle turnover) was equivocal, as a moderate induction of neuronal activity with 25 mM K^+^ did not substantially increase the rate of fluorescence recovery compared with a control group. However, it is important to note that a fraction of GCAMP8-Syb2 sensors are likely located at the surface membrane, complicating straightforward interpretation of fluorescence recovery kinetics in term of vesicle resupply.

It is possible that increasing external Ca^2+^ concentrations would increase the rate of photobleaching with stimulation on ePreCTs, as more fluorophores would be available to be photobleached. Instead, we see similar levels of photobleaching on ePreCTs at higher Ca^2+^ concentrations. This could be due to increased intermixing of Ca^2+^ sensors under high external Ca^2+^ concentrations. If unbleached sensors were mixed into the photobleached vesicle pool, this could decelerate the rate at which Ca^2+^ sensors would have otherwise been photobleached. On the other hand, increasing the extracellular Ca^2+^ concentration increases the effect of photobleaching on baseline Ca^2+^. This result suggests that the Ca^2+^ sensors on vesicle pools that encounter baseline Ca^2+^ do not intermix as well in the presence of elevated external Ca^2+^ concentrations compared with vesicle pools that encounter ePreCTs. These findings further highlight the existence of spatially distinct non-overlapping domains of Ca^2+^ signals within individual terminals. It may be that Ca^2+^ sensors that detect ePreCTs are expressed on vesicle pools in which turnover can be facilitated with activity^58,59^ and that Ca^2+^ sensors that detect baseline Ca^2+^ are expressed on vesicle pools that are refractory to activity-induced turnover.^60,61^

In summary, our experiments address the spatial and functional organization of different Ca^2+^ signals within synaptic terminals. These results strengthen the basis for using photobleaching as a tool to investigate sub-synaptic-level organization as well as the spatial arrangement of other cellular processes. These results also suggest that distinct spatial organization of the same proteins could allow different functions depending on the sub-synaptic localization in which they reside. The spatial compartmentalization of Ca^2+^ signaling could also allow for different proteins to be activated depending on where the Ca^2+^ influx occurs; for instance, vesicles pools being exposed to Ca^2+^ influx of differing kinetics could lead to differences in functional effects. Taken together, these results show that non-overlapping Ca^2+^ signals contribute to distinct forms of neurotransmission and provide further insight into the spatiotemporal properties of Ca^2+^ ions regulating their signaling processes.

### Limitations of the study

While our data demonstrate non-overlapping Ca^2+^ domains within the synapse, the characteristics of the spatiotemporal organization of Ca^2+^ signaling may differ in different neuronal sub-types. Furthermore, to measure Ca^2+^ signals more accurately, we likely need improved approaches to measure presynaptic Ca^2+^. All Ca^2+^ sensors are also Ca^2+^ buffers^16^; as such, the kinetics of the transients we measure are likely distorted compared with the endogenous signal. Ca^2+^ sensors with higher spatial selectivity could better measure the size of different Ca^2+^ domains and whether and how these domain sizes fluctuate in response to increased activity. Further investigation is needed to identify the molecular underpinnings of this strict spatial control.

Our experiments performed at room temperature and the measurement of Ca^2+^ dynamics may slightly differ from measurements performed at more physiological temperatures. For instance, VGCCs as well as synaptic vesicle recycling may show faster kinetics at physiological temperatures, which are all important factors to consider in the context our findings.^62^

Overall, our results expand our understanding of the organization of presynaptic Ca^2+^ and its relationship to neurotransmission. Future experiments and improved optical tools will help delineate the mechanisms and functional consequences of these observations within the wider neurophysiological context.

## STAR★METHODS

### RESOURCE AVAILABILITY

#### Lead contact

Further information and requests for resources and reagents should be directed to and will be fulfilled by the Lead Contact, Ege T. Kavalali (ege.kavalali@vanderbilt.edu).

#### Materials availability

There is one plasmid, pFUGW-GCaMP8s-Syb2, which was generated in this manuscript. All plasmids used in the present manuscript are available for sharing via request to ETK or CSW.

#### Data and code availability

- All data supporting the findings of this study are included as a Source Data file but can also be shared by the lead author Ege T. Kavalali upon request.
- MATLAB codes are available on Github (Github: https://github.com/camilleswang/GCaMP8s-Syb2, Github: https://github.com/camilleswang/iGluSnFR-Analysis) and upon request
- Any additional information required to reanalyze the data reported in this paper is available from the lead contact upon request.

### EXPERIMENTAL MODEL AND STUDY PARTICIPANT DETAILS

#### Animals

For the rat hippocampal cultures, postnatal day 2–3 Sprague-Dawley rats of either sex were used. Pregnant Sprague-Dawley rats (Charles River) were housed individually until they gave birth to a litter and were provided with treats and environmental enrichment. Postnatal day 2–3 littermates were used to prepare primary dissociated neuronal cultures. All animal procedures were performed in accordance with the guide for the care and use of laboratory animals and were approved by the Institutional Animal Care and Use Committee at Vanderbilt University. Health status of the live animals were periodically checked and confirmed by the veterinary staff of animal facilities of the Vanderbilt University.

#### Primary hippocampal culture preparation

Primary hippocampal cultures were generated by dissecting hippocampi from P1–3 Sprague-Dawley rats.^64^ Briefly, dissected hippocampi were washed and treated with 10 mg/mL trypsin and 0.5 mg/mL DNAse at 37°C for 10 min. Tissue was washed again, dissociated with a P1000 tip, and centrifuged at 1400 rpm for 10 min at 4°C. Cells were then resuspended and plated on Matrigel-coated 0 thickness glass coverslips in 24-well plates at a density of 4–6 coverslips per hippocampus.

Plating media contained 10% fetal bovine serum (FBS), 20 mg/L insulin, 2 mM L-glutamine, 0.1 g/L transferrin, 5 g/L D-glucose, 0.2/g NaHCO3 in minimal essential medium (MEM). After 24 h, plating media was exchanged for growth media containing 4 μM cytosine arabinoside (as well as 5% FBS, 0.5 mM L-glutamine, and B27) to inhibit glial proliferation. On days *in vitro* (DIV) 4, growth media was exchanged to a final concentration of 2 μM cytosine arabinoside, and the lentivirus containing our plasmid of interest was added to the culture media. Cultures were kept in the humidified incubators at 37°C and gassed with 95% air and 5% CO2 until DIV 14–21 when plasmid expression was optimal, and experiments were performed.

### METHOD DETAILS

#### Sparse neuron transfection

Neuronal transfections were performed on DIV 7 using a Ca^2+^ phosphate kit (ProFection Mammalian Transfection System, Cat #E1200, Promega), based on a previously described method.^65^ Briefly, A precipitate was formed by mixing the following per each well in a 24-well plate: 1 μg of plasmid DNA, 2 μL of 2 M CaCl2, and 13 μL dH2O. This mixture was then added dropwise to 15 μL of 23 N-2-Hydroxyethylpiperazine-N′−2-Ethanesulfonic Acid (HEPES), while vortexing between drop addition. The precipitate was allowed to form for 15 min. Neuron conditioned media was saved and replaced with MEM and 30 μL of plasmid mixture was added dropwise to each well. Plates were returned to 5% CO2 incubator at 37°C for 30 min. Then cells were washed twice with MEM, after which previously saved conditioned media was added back to each well. Neurons were imaged at DIV 15–18.

#### Live fluorescence imaging

Imaging experiments were done in Tyrode’s buffer. Extracellular Tyrode solution contained (in mM): 150 of NaCl, 4 of KCl, 10 of D-glucose, 10 of HEPES, 2 of MgCl2, 2 of CaCl2 at pH 7.4 and 310–320 mOsm. Imaging buffer also contained 50 μM APV and 10 μM CNQX to prevent recurrent neuronal activity. Fluorescence was recorded using a Nikon Eclipse TE2000-U inverted microscope equipped with a 360 Plan Fluor objective (Nikon, Minato, Tokyo, Japan), a Lambda-DG4 illumination system (Sutter Instruments, Novato, CA, USA) with FITC excitation and emission filters, and an Andor iXon + back illuminated EMCCD camera (Model no. DU-897E-CSO-#BV; Andor Technology, Belfast, UK).

Culture coverslips were randomly assigned for experimental conditions including drug or vehicle groups, and for the order of testing in any given experimental paradigm. Images were acquired at 10 Hz to resolve evoked and spontaneous Ca^2+^ peaks. To induce photobleaching, the neutral density filter within the LAMDA-DG4 illumination system was removed in order to use 100% light intensity. This filter was reintroduced for subsequent live imaging of Ca^2+^ transients after photobleaching. Spontaneous activity was recorded over the course of 10 min. Evoked responses were elicited using a parallel bipolar electrode, delivering 35 mA pulses (0.1ms duration) at 10-s intervals. At the end of each experiment, presynaptic boutons were visualized by delivering a high-frequency electrical stimulation (25 Hz 20 action potentials) or by perfusing 90 mM KCl in Tyrode’s solution.

In the ryanodine experiments, our control group is an equivalent treatment after DMSO (as ryanodine is made in DMSO) in order to account for any artifactual effects of DMSO. DMSO at baseline seems to have a dampening effect on evoked calcium events, which is why there is a wider spread of evoked event detection compared to other experiments. We notice a decrease in iGluSnFR release probability compared to prior experiments (see Wang et al., 2022).^11^ This decrease is not commensurate with the GCaMP decrease, suggesting that DMSO is somehow affecting GCaMP disproportionately.

#### Fluorescence analysis

Imaging data were collected using Nikon Elements Ar software, and the acquired images were subsequently exported to FIJI. On an averaged projection of fluorescence during 90mM K+ or high frequency stimulation, we used a macro script that would detect local maxima at regions of interests (ROIs) of 3 μm diameter, which we found best captured presynaptic bouton’s Ca^2+^ activity and accounted for some drift over time. The measured fluorescence values, as well as image metadata containing treatment and stimulation times, were exported to Microsoft Excel for analysis.

Data were analyzed using an unbiased method based on our previous studies.^7,11^ Given the unbiased method used, blinding was not done for this analysis. Briefly, background was subtracted linearly, and traces were smoothed at up to three points. Spontaneous events were detected using a threshold of 3 standard deviations (SDs) above a moving average (baseline) of 4 s. Evoked events detection was time locked within 0.3 s of an AP delivery, at a threshold of 3 SD above baseline. Parameters including evoked likelihood to stimulation, evoked event amplitudes, spontaneous event frequencies, spontaneous event amplitudes, rise times, and decay times were automatically estimated. All custom MATLAB (Mathworks, Natick, MA, USA) scripts are available on Github and upon request.^66,67^

GCaMP: https://github.com/camilleswang/GCaMP8s-Syb2

GluSnFR: https://github.com/camilleswang/iGluSnFR-Analysis.

### QUANTIFICATION AND STATISTICAL ANALYSIS

Data in graphs were presented as mean ± standard error of the mean (SEM) unless indicated otherwise. Sample sizes were stated in the figure legends and represented as the number of coverslips, unless otherwise indicated. Statistics were done on the averages of coverslips, rather than individual synapses to avoid falsely significant results due to very large sample sizes (the number of synapses and release events can run in the thousands). Individual synaptic values are represented as denoted in several graphs to demonstrate the distribution of values. Sample sizes were based on previous studies in the field of molecular and cellular neuroscience as opposed to using statistical methods prior to experimentation. To ensure reproducibility, each set of experiments were performed across multiple coverslips in at least two sets of cultures.

GraphPad Prism was used to perform the statistical analyses of all other sets of experiments. When normal distribution parameters were met via QQ plot or visualization of the distribution, Welch’s t test was used to compare effects in pairwise datasets obtained from synapses or neurons under distinct conditions. To compare the cumulative histogram of two groups, a Kolmogorov-Smirnov test was used groups. For parametric analysis of multiple comparisons, two-way ANOVA and one-way ANOVA with Tukey post hoc analysis were used. Outliers were identified with Robust regression and Outlier removal (ROUT) method. Significance levels were stated as follows: *p < 0.05, **p < 0.01, ***p < 0.001 and ****p < 0.0001. ns denotes non-significance. See Data S1 for source data, and Tables S2 and S3 in the supplemental file for specific p values.

## Supplementary Material

1

2

3

## Figures and Tables

**Table 1.** Summary of spatial and functional effects of Ca^2+^ signals

Ca^2+^ signal	Ca^2+^ source	Sensitivity to photobleaching at rest	Sensitivity to photobleaching with stimulation	Sensitivity to photobleaching with elevated K^+^	Relationship to neurotransmission
Spontaneous PreCTs	ryanodine-sensitive Ca^2+^ sources	X	X	✓	none
Evoked PreCTs	synchronous VGCC openings	X	✓	✓	evoked glutamate release
Baseline Ca^2+^	stochastic single-channel VGCC opening	✓	✓	✓	spontaneous glutamate release

Sources of Ca^2+^ at presynaptic terminals, as well as their sensitivity to photobleaching and their relationship to neurotransmission. All findings indicate conditions at rest unless otherwise noted (i.e., conditions with stimulation or conditions with elevated K^+^).

**Table:** KEY RESOURCES TABLE

REAGENT or RESOURCE	SOURCE	IDENTIFIER
Chemicals, peptides, and recombinant proteins		
6-Cyano-7-nitroquinoxaline-2,3-dione disodium salt hydrate (CNQX)	Sigma-Aldrich	Catalog #C239
B-27 supplement	Promega	Catalog # 17504–010
Calcium chloride	Sigma Aldrich	Catalog # 21115–250ML
Caffeine	Sigma	Catalog # 1329C0750–5G
Cytosine Arabinoside (Ara-C)	Sigma	Catalog #C6645
D(−)-2-Amino-5-phosphonopentanoic acid (AP-5)	Sigma-Aldrich	Catalog # A8054
D-(+)-Glucose	Sigma	Catalog #G8270–1KG
DMSO	Sigma	Catalog #D2650
DNase I	Sigma-Aldrich	Catalog #D5025
HEPES	Thermo-Fisher	Catalog #H4034–1KG
Insulin	Sigma	Catalog #I0516
L-glutamine	Thermo Fisher	Catalog # 25030–081
Matrigel	Corning	Catalog # 354,230
Magnesium chloride solution	Sigma Aldrich	Catalog # 63069–500ML
MEM: Minimum Essential Medium	Gibco	Catalog # 51200–038
Potassium Chloride	Sigma Aldrich	Catalog #P3911–500G
Ryanodine	Tocris	Catalog # 1329
Sodium bicarbonate	Sigma Aldrich	Catalog # 56297–250G
Sodium chloride	Sigma Aldrich	Catalog #S9888–1KG
Transferrin	Calbiochem	Catalog # 616,420
Trypsin from bovine pancreas	Sigma-Aldrich	Catalog #T9935
Critical commercial assays		
ProFection Mammalian Transfection System	Promega	Catalog #E1200
Deposited data		
MATLAB Code for GCaMP8s-Syb2 Analysis	Generated in lab	https://doi.org/10.5281/zenodo.8274813 [Github]: https://github.com/camilleswang/GCaMP8s-Syb2
MATLAB Code for iGluSnFR Analysis	Generated in lab	https://doi.org/10.5281/zenodo.8274816 [Github]: https://github.com/camilleswang/iGluSnFR-Analysis
Experimental models: Organisms/strains		
Sprague-Dawley rats, CD1 (Sprague-Dawley postnatal pups P2–3, M and F)	Charles River	Strain code: 400
Recombinant DNA		
Plasmid: pFUW-GCaMP8s-Syb2	Generated in lab	N/A
Plasmid: pCI syn iGluSnFR	Helassa et al.^27^	pCI syn iGluSnFR; Addgene Cat #106123
Software and algorithms		
Prism 8	Prism 8	https://www.graphpad.com/
Fiji	Schindelin et al.^63^	https://imagej.net/software/fiji/downloads
MATLAB. (2018). 9.7.0.1190202 (R2019b).	Natick, Massachusetts: The MathWorks Inc.	https://www.mathworks.com/products/matlab.html?s_tid=hp_products_matlab
Nikon Elements Viewer 4.50	Nikon: NIS Elements Viewer	https://www.microscope.healthcare.nikon.com/products/software/nis-elements/viewer
Other		
35mm Dish | No. 1.5 Gridded Coverslip | 14 mm Glass Diameter	MatTek	Catalog #P35G-1.5-14-CGRD
Cover Glasses 0.09–0.12 mm (No. 0) Circles 12mm	Carolina Biological	Catalog # 633009
24 well plate	Fisher Scientific	Catalog # Greiner 662160
Andor iXon + back illuminated EMCCD camera	Andor Technology	Model no. DU-897E-CSO-#BV
Heracell^™^ 150i and 240i CO2 Incubators with Stainless-Steel Chambers	Thermo Scientific	Mfr. No.51026282
Lambda-DG4 illumination system	Sutter Instruments	Model: DG-4
Nikon Eclipse TE2000-U inverted microscope equipped with a 360 Plan Fluor objective	Nikon	SKU: 16436
Stimulus Isolator	World Precision Instruments	Model # A385
Vapor Pressure Osmometer	Viescor	Model # 5520

## Highlights

- Evoked PreCTs, spontaneous PreCTs, and baseline Ca^2+^ derive from distinct Ca^2+^ sources
- These Ca^2+^ signals originate from spatially non-overlapping domains within single synapses
- The three distinct Ca^2+^ signals drive specific modes of neurotransmission
- Synaptic vesicle turnover occurs across presynaptic terminals within minutes

## INCLUSION AND DIVERSITY

One or more of the authors of this paper self-identifies as a gender minority in their field of research. One or more of the authors of this paper self-identifies as a member of the LGBTQIA+ community. We support inclusive, diverse, and equitable conduct of research.
