# Supplementary material for: Spatially non-overlapping Ca2+ signals drive distinct forms of neurotransmission
Source: Cell Rep. Author manuscript; Available in PMC 2024 Feb 5. (PMC10842353; doi:10.1016/j.celrep.2023.113201)
Supplement: 1 [file NIHMS1941885-supplement-1.pdf]

Cell Reports, Volume *42*

Supplemental information

Spatially non-overlapping Ca^{2+} signals drive distinct forms of neurotransmission

Camille S. Wang, Lisa M. Monteggia, and Ege T. Kavalali

SUPPLEMENTARY FIGURES

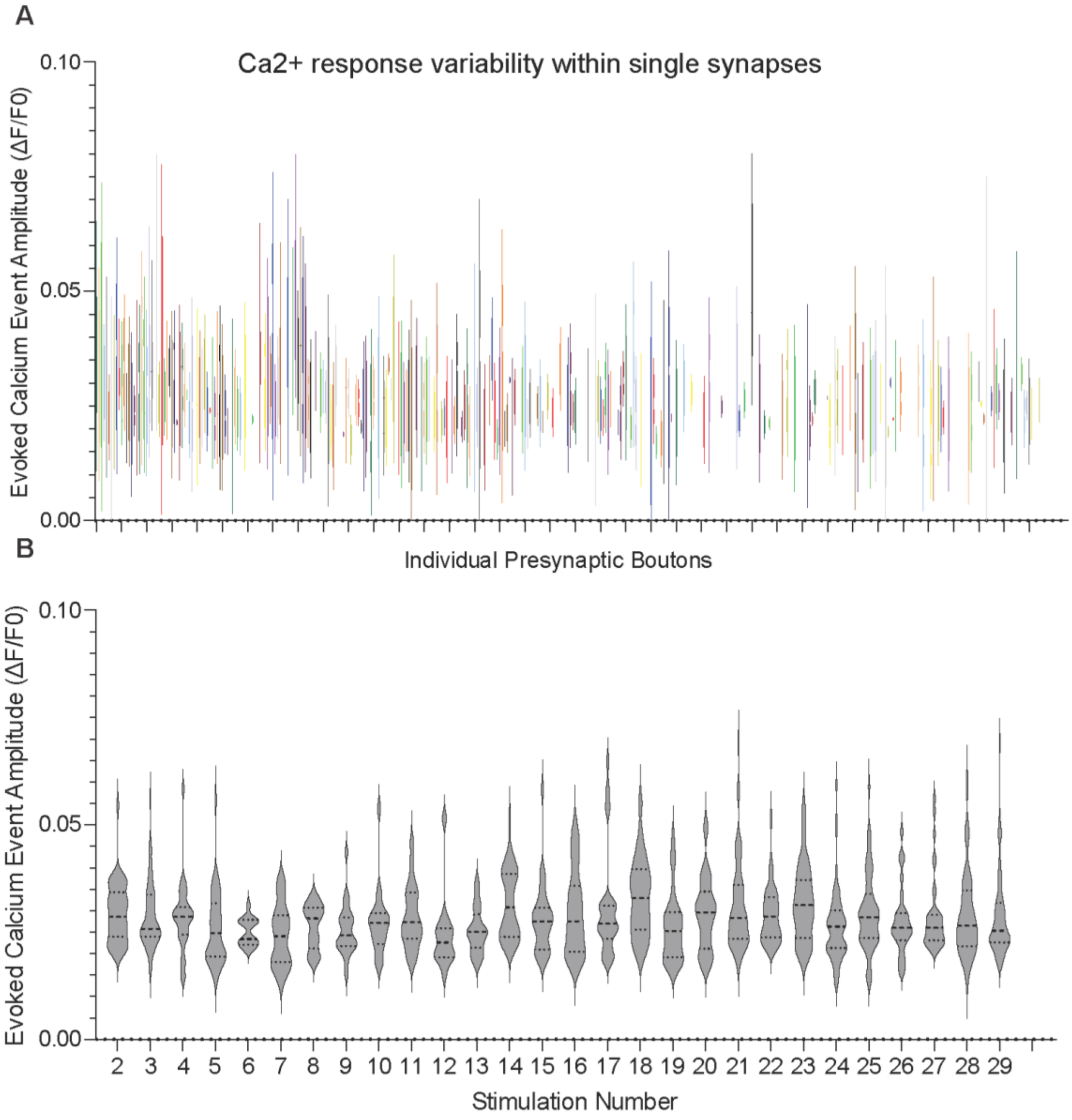

Supplemental Figure S1. Individual axonal boutons demonstrate unique ePreCT amplitudes

(A) Individual axonal boutons and their respective Ca^{2+} amplitudes upon stimulation

(B) When averaged, each stimulation elicits similar ePreCT amplitudes, suggesting the individual differences are not related to stimulation failure.

These data were plotted from 2 biological and 8 technical replicates.

Related to Figure 1.

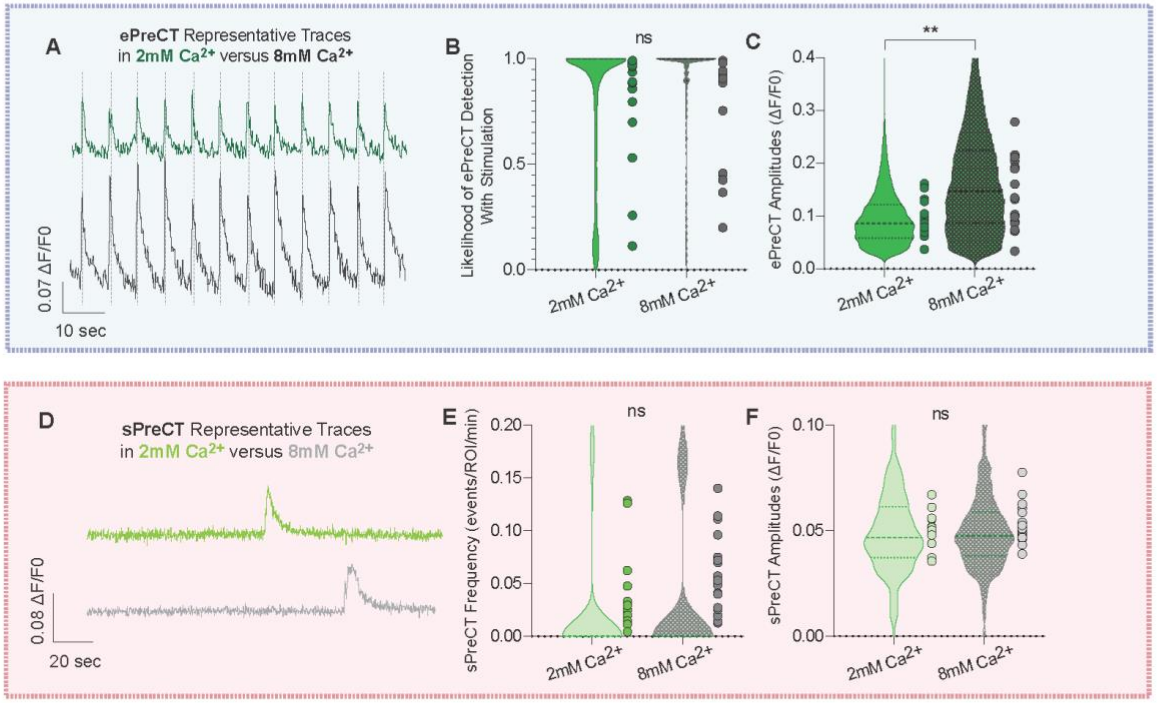

Supplemental Figure S2. Presynaptic Ca^{2+} transients in 2mM Ca^{2+} or 8mM Ca^{2+}

(A) Representative ePreCT traces in different Ca^{2+} concentrations

(B – C) Comparison of ePreCT likelihood of event detection to stimulation (B) and event amplitudes (C) in 2mM Ca^{2+} (N=12 coverslips) versus 8mM Ca^{2+} (N=24 coverslips). Welch's t-test.

(D) Representative sPreCT traces in different Ca^{2+} concentrations

(E – F) Comparison of sPreCT frequencies (B) and event amplitudes (C) in 2mM Ca^{2+} versus 8mM Ca^{2+}. Welch's t-test.

Graphs are mean ± SEM. Significance reported as $^{**}p < 0.01$. NS denotes non-significance. Related to Figure 1.

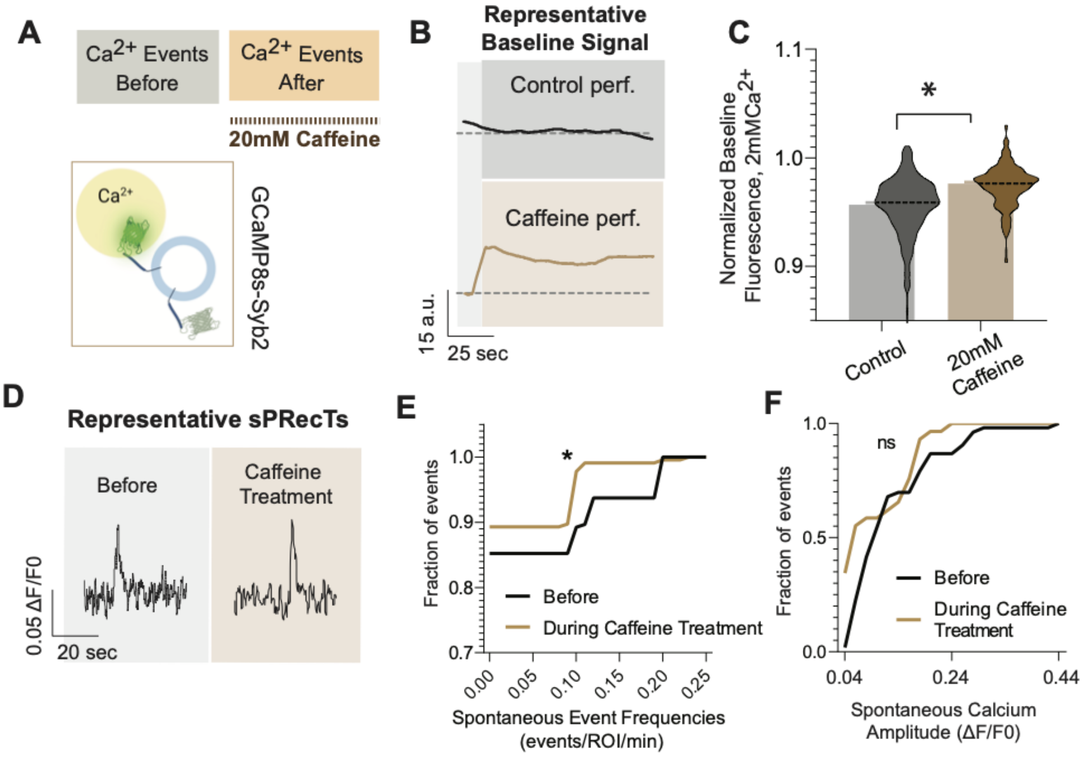

Supplemental Figure S3. Effect of caffeine perfusion on presynaptic Ca^{2+} signals

(A) Experimental paradigm of caffeine perfusion

(B) Representative traces of baseline Ca^{2+} signals after control versus caffeine perfusion

(C) Effect of 20mM caffeine perfusion on baseline Ca^{2+} fluorescence compared to a control treatment. Violin plot represents synaptic values. Welch's t-test.

(D) Representative PreCTs before and during caffeine treatment

(E) Cumulative histogram of sPreCT frequencies before and during caffeine treatment. Welch's t-test.

(F) Cumulative histogram of sPreCT amplitudes before and during caffeine treatment. Welch's t-test.

Graphs are mean ± SEM. Significance reported as $^*p < 0.05$. NS denotes non-significance. Two biological replicates were performed with similar results, though only one biological replicate and 5 technical replicates are shown in this graph.

Related to Figure 3

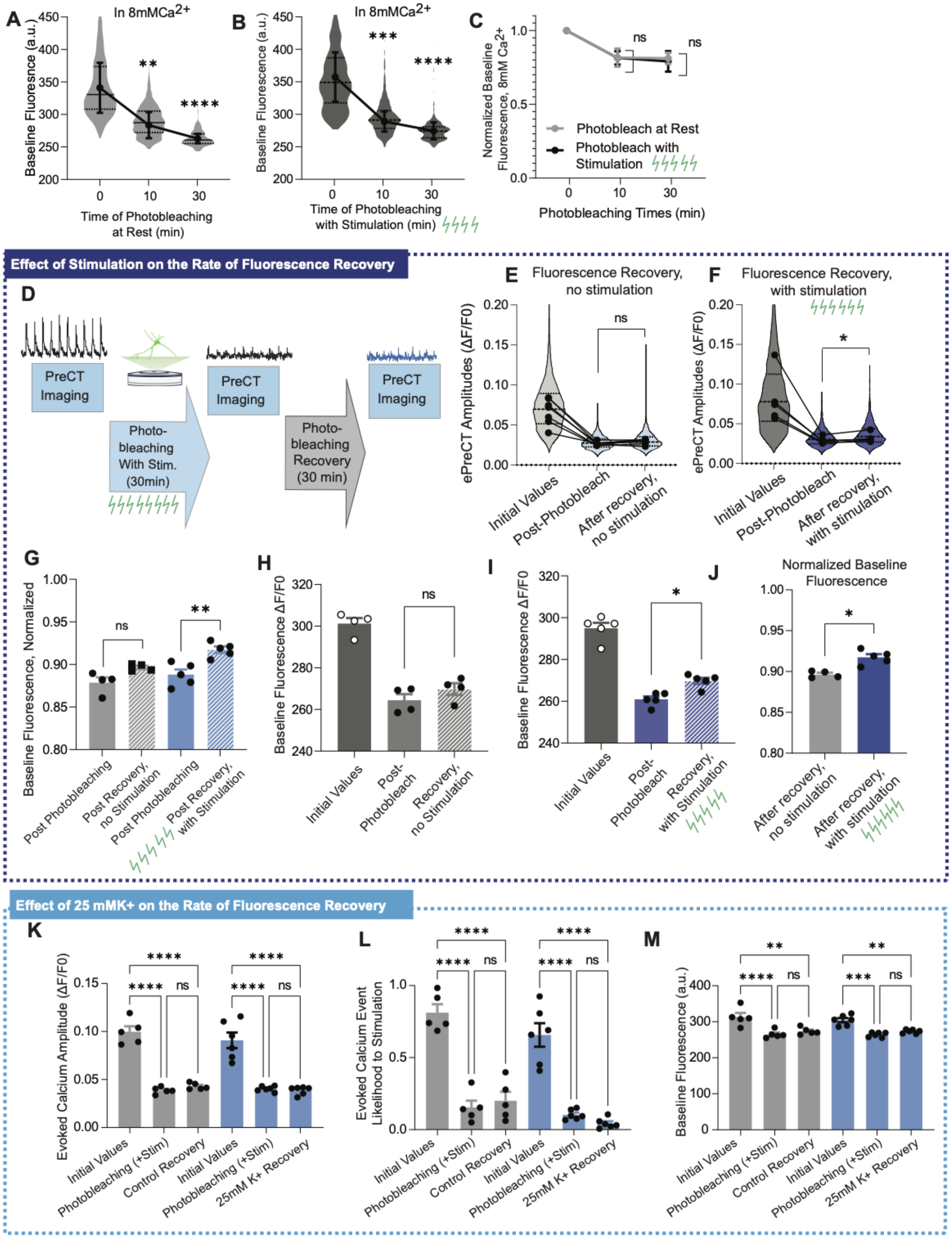

Supplemental Figure S4. Photobleaching baseline Ca^{2+} signal in 8mM Ca^{2+} external concentrations

(A) Photobleaching at rest of baseline Ca^{2+} signal in 8mMCa Ca^{2+}. N=7 coverslips for 10 minutes of photobleaching, N=6 coverslips for 30 minutes of photobleaching. One-way ANOVA.
(B) Photobleaching with stimulation of baseline Ca^{2+} signal in 8mMCa Ca^{2+}. N=7 coverslips for 10 minutes of photobleaching, N=6 coverslips for 30 minutes of photobleaching. One-way ANOVA.
(C) Comparison of photobleaching at rest versus photobleaching with stimulation of baseline Ca^{2+} signal in 8mM Ca^{2+}. Two-way ANOVA.
Graphs are mean ± SEM. Significance reported as **p < 0.01, ***p < 0.001, and ****p < 0.0001. NS denotes non-significance.
(D) Experimental paradigm of fluorescence recovery for 30 minutes with and without stimulation
(E) Effect of fluorescence recovery for 30 minutes, no applied stimulation during the recovery. N=7 coverslips. One-way ANOVA. Reshown from Figure 7 for context.
(F) Effect of fluorescence recovery for 30 minutes, with 0.5Hz stimulation applied during the recovery period. N=5 coverslips. One-way ANOVA.
(G) Comparison of normalized baseline fluorescence after photobleaching with stimulation, followed by recovery with and without stimulation. One-way ANOVA.
(H) Effect of no stimulation on fluorescence recovery of baseline signal for 30 minutes. Paired t-test.
(I) Effect of 0.5 Hz stimulation on fluorescence recovery of baseline signal for 30 minutes. Paired t-test.
(J) Comparison of fluorescence recovery of baseline Ca2+ signal with versus without stimulation. Welch's t-test.
(K) Effect of 25mM K+ during the fluorescence recovery period (30 min) compared to control treatment on evoked calcium amplitude. One-way ANOVA.
(L) Effect of 25mM K+ during the fluorescence recovery period (30 min) compared to control treatment on evoked calcium likelihood to stimulation. One-way ANOVA.
(M) Effect of 25mM K+ during the fluorescence recovery period (30 min) compared to control treatment on baseline fluorescence. One-way ANOVA.
Related to Figure 7

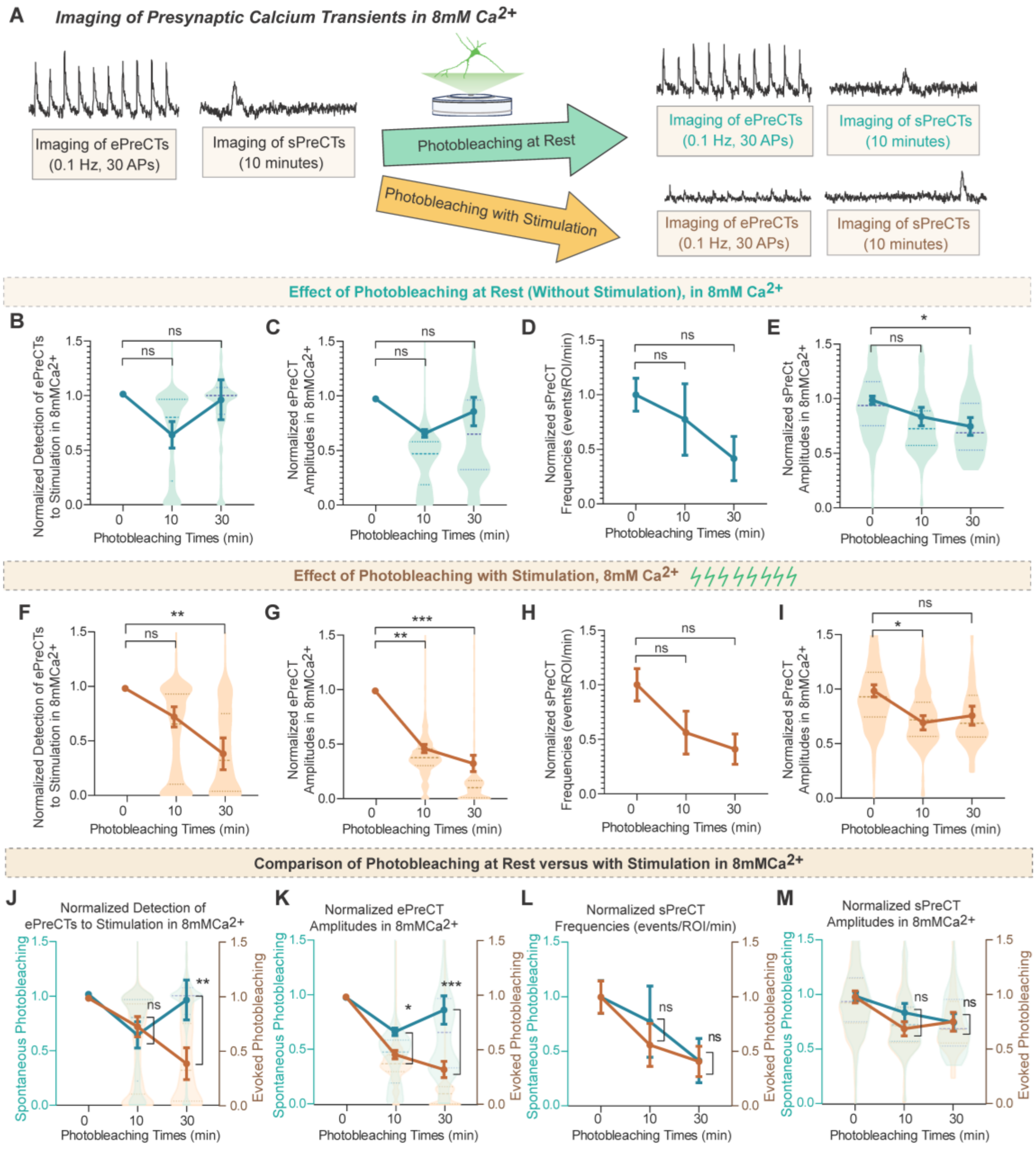

Supplemental Figure 5: Effects of photobleaching in 8mMCa^{2+}

(A) Experimental paradigm of photobleaching in 8mM Ca^{2+}

(B – E) Effect of photobleaching at rest on ePreCT (B – C) and sPreCTs (D – E) in 8mM Ca^{2+}. N=7 coverslips for 10 minute photobleaching, N=6 coverslips for 30 minute photobleaching. One-way ANOVA.

(F – I) Effect of photobleaching with stimulation on ePreCT (F – G) and sPreCTs (H – I) in 8mM Ca^{2+}. N=6 coverslips for 10 minute photobleaching, N=6 coverslips for 30 minute photobleaching. One-way ANOVA.

(J – M) Comparison of photobleaching at rest versus with stimulation in 8mM Ca^{2+}

Graphs are mean ± SEM. Significance reported as *p < 0.05, **p < 0.01, and ***p < 0.001. NS denotes non-significance.

Related to Figure 7.

Table S2: Main Figures P-Values

**Figure 1**	**p-value label**	**p-value numbers**	**Description**	**Statistical Test**
1L	***	0.0076	Amplitude	Welch's t-test
1M	***	0.0001	Rise Time	
1N	ns	0.3444	Decay Time	
**Figure 2**	**p-value label**	**p-value numbers**	**Description**	**Statistical Test**
2B	****	<0.0001	Baseline vs. + Cono Aga	1-way AVOVA
	****	<0.0001	Baseline vs. + Cono Aga SNX	
	ns	0.5685	+ Cono Aga vs. + Cono Aga SNX	
2C	****	<0.0001	Baseline vs. + Cono Aga	1-way ANOVA
	****	<0.0001	Baseline vs. + Cono Aga SNX	
	ns	0.6049	+ Cono Aga vs. + Cono Aga SNX	
2E	ns	0.8796	Baseline vs. + Cono Aga	1-way ANOVA
	ns	0.7993	Baseline vs. + Cono Aga SNX	
	ns	0.9891	+ Cono Aga vs. + Cono Aga SNX	
2F	ns	0.6354	Baseline vs. + Cono Aga	1-way ANOVA
	ns	0.9825	Baseline vs. + Cono Aga SNX	
	ns	0.5261	+ Cono Aga vs. + Cono Aga SNX	
2H		>0.9999	Baseline	2-way ANOVA
		0.0118	Cono Aga	
		0.0012	Cono Aga SNX	
2J	*	0.029	Baseline vs. + Cono Aga	1-way ANOVA
	*	0.0229	Baseline vs. + Cono Aga SNX	
	ns	0.092	+ Cono Aga vs. + Cono Aga SNX	
2K	ns	0.1099	Before vs 2nd	1-way ANOVA
	ns	0.0634	Before vs 3rd	
	ns	0.3542	2nd vs 3rd	
2L	ns	>0.9999	Before	2-way ANOVA
	*	0.0202	Cono Aga	
	*	0.0314	Cono Aga SNX	
2N	****	<0.0001	Baseline vs. + Cono Aga	1-way ANOVA
	****	<0.0001	Baseline vs. + Cono Aga SNX	
	ns	>0.9999	+ Cono Aga vs. + Cono Aga SNX	
2O	**	0.0011	Baseline vs. + Cono Aga	1-way ANOVA

	***	0.0004	Baseline vs. + Cono Aga SNX	
	ns	0.6709	+ Cono Aga vs. + Cono Aga SNX	
2P	ns	0.9588	Baseline vs. + Cono Aga	1-way ANOVA
	ns	0.9914	Baseline vs. + Cono Aga SNX	
	ns	0.9875	+ Cono Aga vs. + Cono Aga SNX	
**Figure 3**	**p-value label**	**p-value numbers**	**Description**	**Statistical Test**
3B	*	0.0142	sPreCT Frequency	Welch's t-test
3C	ns	0.8756	sPreCT Amplitudes	Paired t-test
3E	ns	0.2478	ePreCT Likelihood	Welch's t-test
3F	ns	0.7852	ePreCT Amplitues	Welch's t-test
3G	ns	>0.9999	Before	2-way ANOVA
	ns	>0.9999	After Ryanodine or Control	
3I	ns	0.6924	Standard Deviation	Welch's t-test
3K	ns	0.426	Spontaneous Glutamate Freq	Welch's t-test
3L	ns	0.3404	Spontaneous Glutamate Amp	Welch's t-test
3M	ns	0.3595	Est Release Probability	Welch's t-test
3N	ns	0.3535	Evoked Event Amp	Welch's t-test
3O	****	<0.0001	Event Frequencies	Welch's t-test
**Figure 4**	**p-value label**	**p-value numbers**	**Description**	**Statistical Test**
4C	ns	0.9924	0 vs 10 min	1-way ANOVA
	ns	0.6989	0 vs 30 min	
	ns	0.8191	10 min vs 30 min	
4D	ns	0.6236	0 vs 10 min	1-way ANOVA
	ns	0.6041	0 vs 30 min	
	ns	0.9996	10 min vs 30 min	
4E	ns	0.8134	0 vs 10 min	1-way ANOVA
	ns	0.816	0 vs 30 min	
	ns	0.4089	10 min vs 30 min	
4F	ns	0.9699	0 vs 10 min	1-way ANOVA
	ns	0.9591	0 vs 30 min	
	ns	0.876	10 min vs 30 min	
4G	*	0.0289	0 vs 10 min	1-way ANOVA
	**	0.0024	0 vs 30 min	
	ns	0.4671	10 min vs 30 min	
4H	*	0.0184	0 vs 10 min	1-way ANOVA
	***	0.0009	0 vs 30 min	
	ns	0.5419	10 min vs 30 min	
4I	ns	0.9595	0 vs 10 min	1-way ANOVA

	ns	0.8751	0 vs 30 min	
	ns	0.9761	10 min vs 30 min	
4J	ns	0.9567	0 vs 10 min	1-way ANOVA
	ns	0.5845	0 vs 30 min	
	ns	0.7724	10 min vs 30 min	
**Figure 5**	**p-value label**	**p-value numbers**	**Description**	**Statistical Test**
5B	*	0.0165	sPreCT Frequencies	Paired t-test
5C	*	0.0106	sPreCT Amplitudes	Paired t-test
5D	****	<0.0001	ePreCT Likelihood	Paired t-test
5E	****	<0.0001	ePreCT Amplitudes	Paired t-test
5F	**	0.0081	Baseline Fluorescence	Welch's t-test
**Figure 6**	**p-value label**	**p-value numbers**	**Description**	**Statistical Test**
6B	ns	>0.9999	0 min	2-way ANOVA
	**	0.0034	10 min	
	****	<0.0001	30 min	
6D	ns	>0.9999	0 min	2-way ANOVA
	***	0.0001	10 min	
	****	<0.0001	30 min	
6E	ns	>0.9999	0 min	2-way ANOVA
	ns	0.2101	10 min	
	ns	0.48	30 min	
6F	**	0.0056		Welch's t-test
6G	ns	>0.9999	0 min	2-way ANOVA
	**	0.001	10 min	
	*	0.0299	30 min	
6J	***	0.0001	Before vs. 10 min	
	****	<0.0001	Before vs. 30 min	
	ns	0.7719	10 min vs. 30 min	
6K	****	<0.0001	Before vs. 10 min	
	****	<0.0001	Before vs. 30 min	
	ns	0.9785	10 min vs. 30 min	
6L	ns	0.6157		2-way ANOVA
**Figure 7**	**p-value label**	**p-value numbers**	**Description**	**Statistical Test**
7B	ns	>0.9999	0 min	1-way ANOVA
	ns	0.5522	10 min	
	ns	0.9988	30 min	
7C	ns	>0.9999	0 min	1-way ANOVA

	ns	0.9999	10 min	
	ns	0.9827	30min	
7E	ns	0.0787		Paired t-test
7F	*	0.0167	Paired t test	Paired t-test
7H	****	<0.0001	Initial means vs. Post-Bleach means	1-way ANOVA
	****	<0.0001	Initial means vs. 1hr means	
	ns	0.0713	Initial means vs. 5hr means	
	ns	0.8451	Initial means vs. 14hr means	
7I	****	<0.0001	Initial means vs. Post-Bleach means	1-way ANOVA
	ns	0.8707	Initial means vs. 1hr means	
	ns	0.388	Initial means vs. 5hr means	
	ns	0.9988	Initial means vs. 14hr means	
7J	****	<0.0001	Initial means vs. Post-Bleach means	1-way ANOVA
	***	0.0002	Initial means vs. 1hr means	
	ns	0.9993	Initial means vs. 5hr means	
	ns	0.1522	Initial means vs. 14hr means	

Table S3: Supplementary Figures P-Values

**Figure S2**	**p-value label**	**p-value numbers**	**Description**	**Statistical Test**
S2B	ns	0.8751		Welch's t-test
S2C	**	0.006		Welch's t-test
S2E	ns	0.0633		Welch's t-test
S2F	ns	0.4431		Welch's t-test
**Figure S3**	**p-value label**	**p-value numbers**	**Description**	**Statistical Test**
S3C	*	0.0207		2-way ANOVA
S3E	*	0.0135		Welch's t-test
S3F	ns	0.3313		Welch's t-test
**Figure S4**	**p-value label**	**p-value numbers**	**Description**	**Statistical Test**
S4A	**	0.0012	0 min vs. 10 min	1-way ANOVA
	****	<0.0001	0 min vs. 30 min	
	ns	0.4391	10 min vs. 30 min	
S4B	**	0.0083	0 min vs. 10 min	1-way ANOVA
	**	0.0022	0 min vs. 30 min	
	ns	0.9442	10 min vs. 30 min	
S4C	ns	>0.9999	0 min vs. 10 min	1-way ANOVA
	ns	0.9998	0 min vs. 30 min	
	ns	0.8149	10 min vs. 30 min	

S4E	ns	0.0787		Paired t-test
S4F	*	0.0167		Paired t-test
S4G	ns	0.1193		1-way ANOVA
	**	0.0028		
S4H	ns	0.4047		1-way ANOVA
S4I	*	0.0167		1-way ANOVA
S4K	****	<0.0001	Initial vs. Post-Bleach	1-way ANOVA
	****	<0.0001	Initial vs. Control Recovery	
	ns	0.9855	Post-Bleach vs. Control Recovery	
	****	<0.0001	Initial vs. Post-Bleach	
	****	<0.0001	Initial vs. 25mM K+ Recovery	
	ns	>0.9999	Post-Bleach vs. 25mM K+ Recovery	
S4L	****	<0.0001	Initial vs. Post-Bleach	
	****	<0.0001	Initial vs. after Control Recovery	
	ns	0.9887	Post-Bleach vs. Control Recovery	
	****	<0.0001	Initial vs. Post-Bleach	
	****	<0.0001	Initial vs. 25mM K+ Recovery	
	ns	0.6616	Post-Bleach vs. 25mM K+ Recovery	
S4M	****	<0.0001	Initial vs. Post-Bleach	
	**	0.001	Initial vs. after Control Recovery	
	ns	0.9089	Post-Bleach vs. Control Recovery	
	****	<0.0001	Initial vs. Post-Bleach	
	***	0.0004	Initial vs. 25mM K+ Recovery	
	ns	0.9272	Post-Bleach vs. 25mM K+ Recovery	
**Figure S4**	**p-value label**	**p-value numbers**	**Description**	**Statistical Test**
S4B	ns	0.0893	0 min vs. 10 min	1-way ANOVA
	ns	0.955	0 min vs. 30 min	
S4C	ns	0.4138	0 min vs. 10 min	1-way ANOVA
	ns	0.8833	0 min vs. 30 min	
S4D	ns	0.6873	0 min vs. 10 min	1-way ANOVA
	ns	0.1384	0 min vs. 30 min	
S4E	ns	0.1639	0 min vs. 10 min	1-way ANOVA
	*	0.0194	0 min vs. 30 min	
S4F	ns	0.1776	0 min vs. 10 min	1-way ANOVA
	**	0.002	0 min vs. 30 min	
S4G	**	0.0069	0 min vs. 10 min	1-way ANOVA
	***	0.0009	0 min vs. 30 min	
S4H	ns	0.1739	0 min vs. 10 min	1-way ANOVA

	ns	0.0514	0 min vs. 30 min	
S4I	*	0.0115	0 min vs. 10 min	1-way ANOVA
	ns	0.0505	0 min vs. 30 min	
S4J	ns	0.8335	0 min vs. 10 min	2-way ANOVA
	**	0.0066	0 min vs. 30 min	
S4K	*	0.0479	0 min vs. 10 min	2-way ANOVA
	****	<0.0001	0 min vs. 30 min	
S4L	ns	0.9004	0 min vs. 10 min	2-way ANOVA
	ns	>0.999	0 min vs. 30 min	
S4M	ns	0.3819	0 min vs. 10 min	2-way ANOVA
	ns	0.991	0 min vs. 30 min	
